# Supplementary material for: Elucidating the structure-stability relationship of Cu single-atom catalysts using operando surface-enhanced infrared absorption spectroscopy
Source: Nat Commun. 2023 Dec 14;14:8311. doi: 10.1038/s41467-023-44078-1 (PMC10721631; doi:10.1038/s41467-023-44078-1)
Supplement: Supplementary file 1 — supplementary information [file 41467_2023_44078_MOESM1_ESM.pdf]

Supplementary Information

Elucidating the Structure-Stability Relationship of Cu Single-Atom Catalysts Using *Operando* Surface-Enhanced Infrared Absorption Spectroscopy

Li Zhang,[1,2] Xiaoju Yang,[1,2] Qing Yuan,[1,2] Zhiming Wei,[3] Jie Ding,[3] Tianshu Chu,[4] Chao Rong,[4] Qiao Zhang,[3] Zhenkun Ye,[1,2] Fu-Zhen Xuan,[4] Yueming Zhai,[3] Bowei Zhang,[4,*] Xuan Yang[1,2,*]

[1]Key Laboratory of Material Chemistry for Energy Conversion and Storage, Huazhong University of Science and Technology, Wuhan 430074, China

[2]Hubei Key Laboratory of Material Chemistry and Service Failure, School of Chemistry and Chemical Engineering, Huazhong University of Science and Technology, Wuhan 430074, China

[3]The Institute for Advanced Studies, Wuhan University, Wuhan 430072, China

[4]Shanghai Key Laboratory of Intelligent Sensing and Detection Technology, Key Laboratory of Pressure Systems and Safety of Ministry of Education, School of Mechanical and Power Engineering, East China University of Science and Technology, Shanghai 200237, China

[*]Corresponding authors: boweiz@ecust.edu.cn and xuanyang@hust.edu.cn

Contents

1. Morphology and Structure Characterizations of the Cu/C_3N_4 Catalysts

2. Scheme of the Spectroelectrochemical Cell for *Operando* SEIRAS Measurements

3. *Operando* SEIRAS Measurements of Cu Species in the Cu/C_3N_4 Catalysts

4. Procedure for the Quantification of Evolution Rate

5. Morphology and Structure Characterizations of the Cu/C_3N_4 SACs post CO_2RR

6. Morphology and Structure Characterizations of the CuPc, Cu-NC, and Cu-SNC Catalysts

7. *Operando* SEIRAS Measurements of the CuPc, Cu-NC, and Cu-SNC Catalysts

8. Morphology Characterizations of the CuPc, Cu-NC, and Cu-SNC Catalysts Post CO_2RR

9. Evolution Rates of the CuPc, Cu-NC, and Cu-SNC Catalysts

10. CO_2RR Performance of Cu SACs

11. Cu Content and EXAFS Fitting Parameters for Cu SACs

1. Morphology and Structure Characterizations of the Cu/C_3N_4 Catalysts

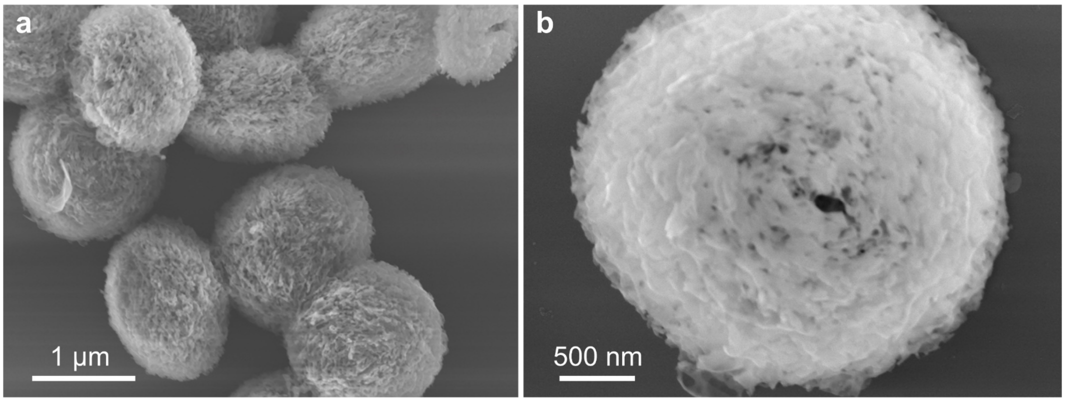

Supplementary Fig. 1. SEM images of the Cu/C_3N_4 SACs at different magnifications before the CO_2RR (**a** and **b**).

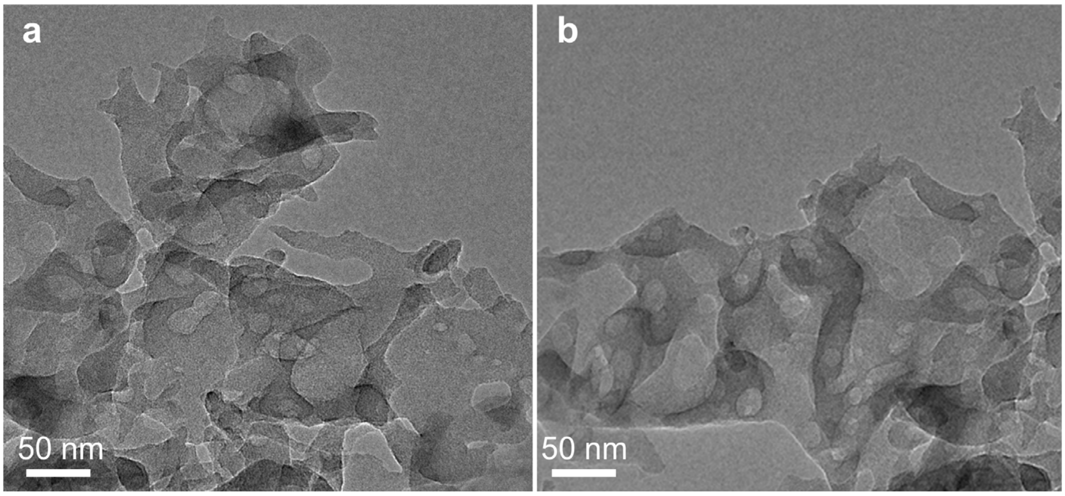

Supplementary Fig. 2. TEM images of the Cu/C_3N_4 SACs before the CO_2RR (**a** and **b**). There are no Cu nanoparticles in the catalysts.

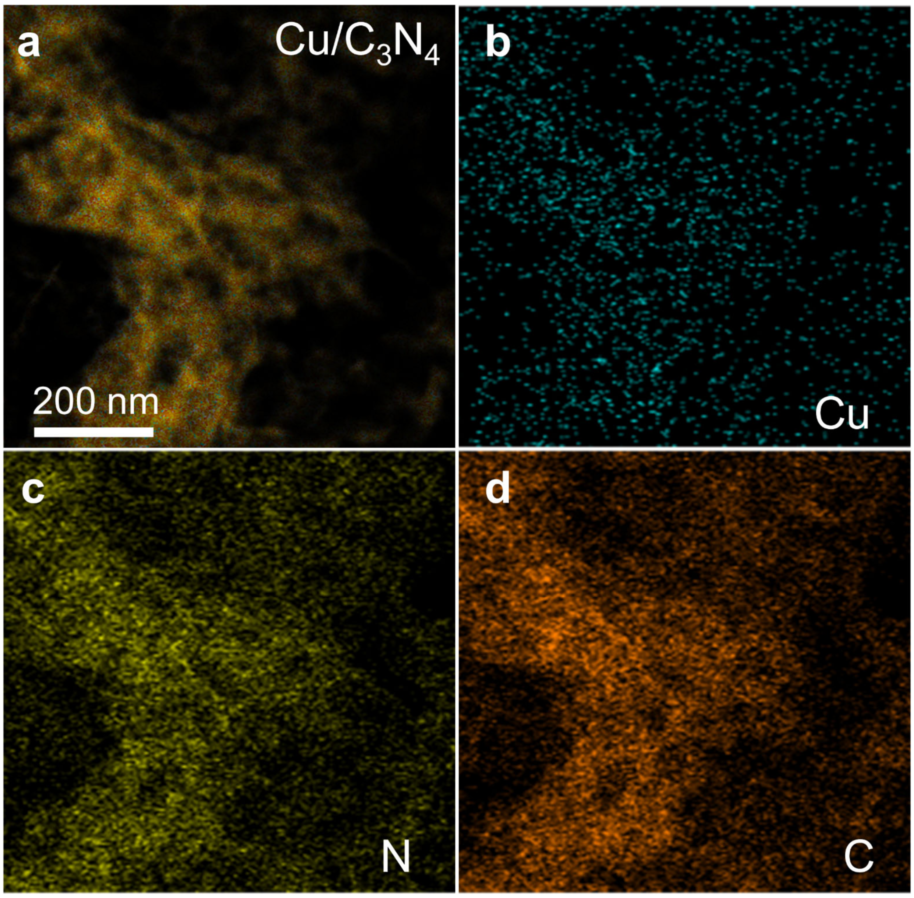

Supplementary Fig. 3. (**a**) HAADF-STEM image of the Cu/C_3N_4 SACs. **b-d** EDX mapping of elemental distributions for (**b**) Cu, (**c**) N, and (**d**) C in the Cu/C_3N_4 SACs.

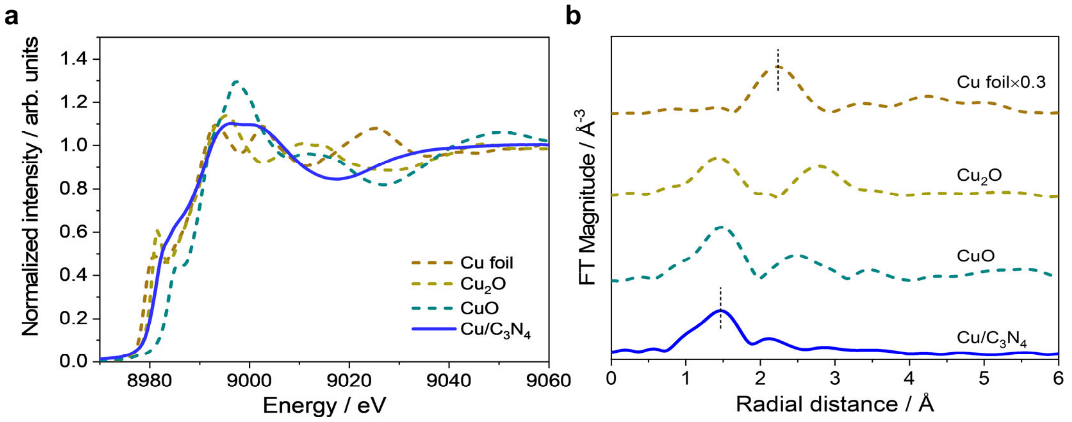

Supplementary Fig. 4. Normalized XANES (**a**) and FT-EXAFS (**b**) spectra of the Cu/C_3N_4 SACs with Cu foil, Cu_2O, and CuO as references. Note that the intensity of the EXAFS spectrum of Cu foil is amplified by 0.3 times.

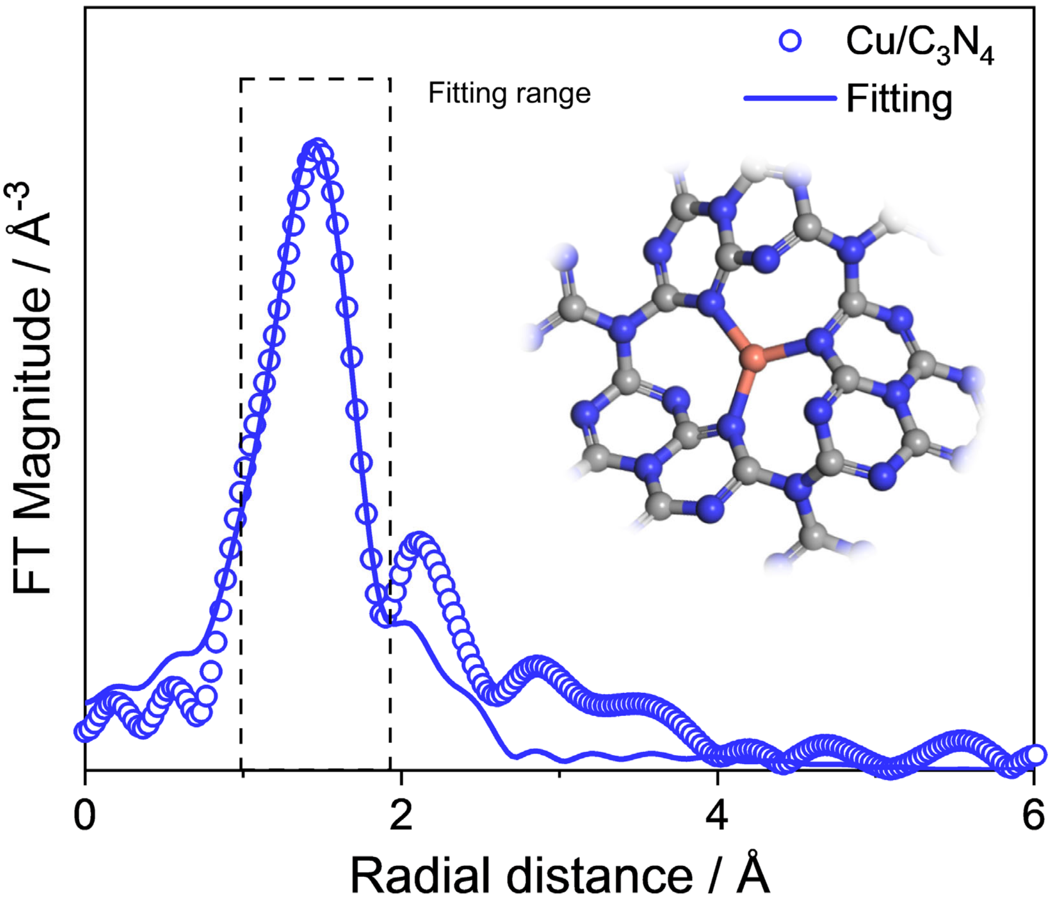

Supplementary Fig. 5. The FT-EXAFS fitting profile of the Cu K-edge peak in the Cu/C_3N_4 SACs in the range of 1.0−1.9 Å.

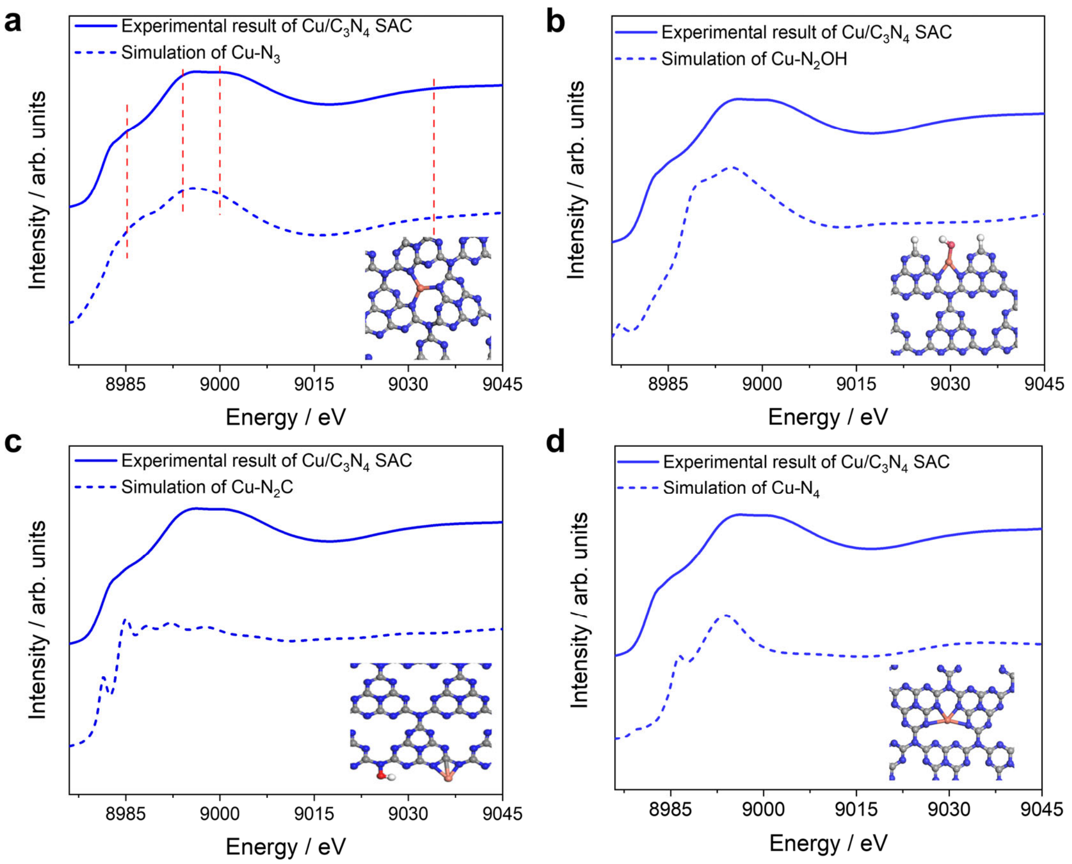

Supplementary Fig. 6. Simulated XANES spectra for four possible geometrical structures of Cu/C_3N_4 catalysts. (**a**) Cu-N_3. (**b**) Cu-N_2OH. (**c**) Cu-N_2C. (**d**) Cu-N_4.

2. Scheme of the Spectroelectrochemical Cell for *Operando* SEIRAS Measurements

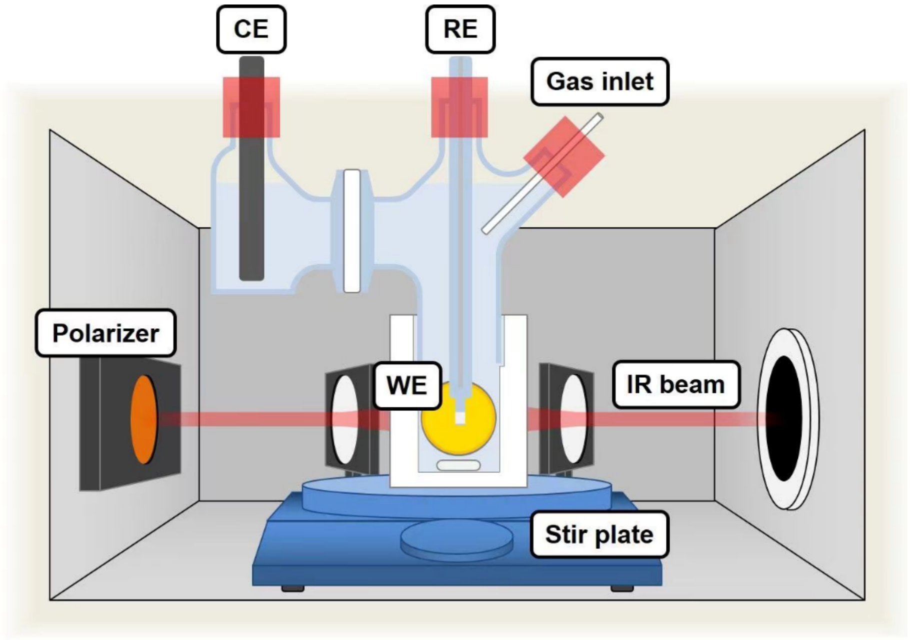

Supplementary Fig. 7. Schematic of the stirred spectroelectrochemical cell for *operando* ATR-SEIRAS.[1]

3. *Operando* SEIRAS Measurements of Cu Species in the Cu/C_3N_4 Catalysts

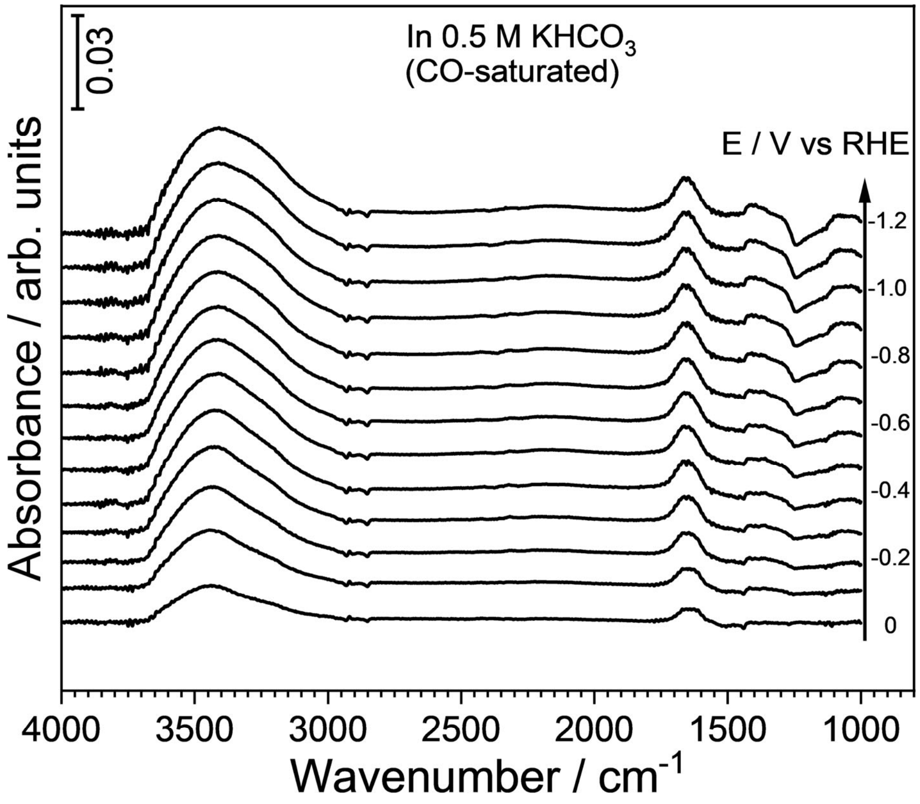

Supplementary Fig. 8. Potential-dependent SEIRA spectra on the surfaces of C_3N_4 catalysts in the presence of CO-saturated 0.5 M $KHCO_3$ solution.

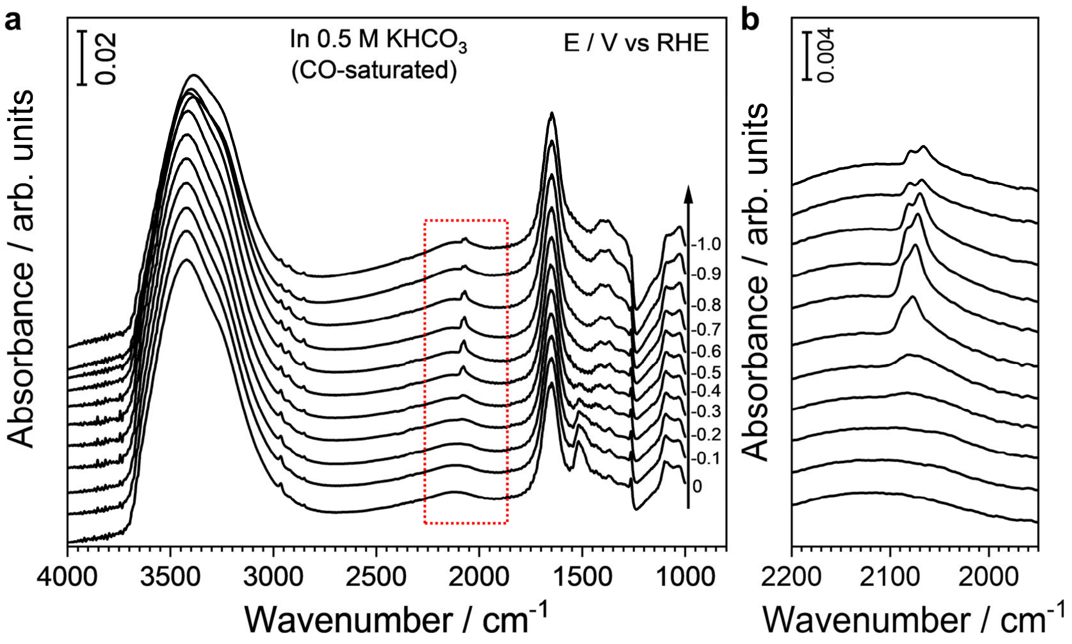

Supplementary Fig. 9. (**a**) Potential-dependent SEIRA spectra collected on Cu films in CO-saturated 0.5 M $KHCO_3$ solution (reference spectrum collected at the open circuit potential). (**b**) The corresponding SEIRA spectra in the range of 1800–2200 cm^{-1} (orange rectangle in **a**). The band at around 2086 $cm^{-1}$and 2074 cm^{-1} are attributed to CO adsorption on Cu step and Cu terrace sites, respectively.

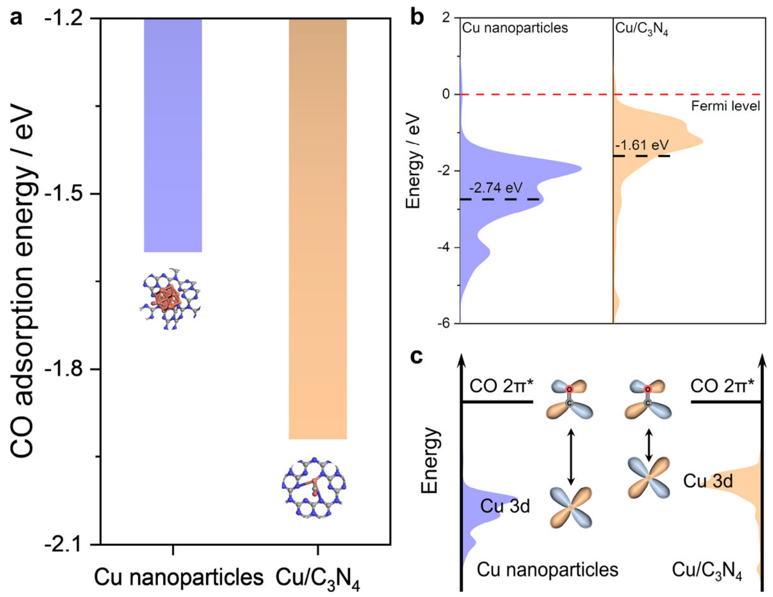

Supplementary Fig. 10. (**a**) CO adsorption energy for Cu/C_3N_4 SACs and Cu nanoparticles. (**b**) Calculated projected density of state (PDOS) of Cu 3d orbital. (**c**) Schematic illustration showing strong electron backdonation in the Cu/C_3N_4 SACs.

The d-band center of the Cu/C_3N_4 catalysts locates at −1.61 eV, which is significantly higher than that of Cu nanoparticles (−2.74 eV, Supplementary Fig. 10b). Therefore, the CO adsorption energy is much lower on Cu singles sites, leading to stronger bonding interactions between CO and Cu single sites.[2] Furthermore, the unoccupied electron of $2\pi^*$ orbital of CO could partially shift under the Fermi levels and be occupied via backdonation of Cu 3d electron,[3] leading to enhanced bond strength of Cu-CO (Supplementary Fig. 10c).

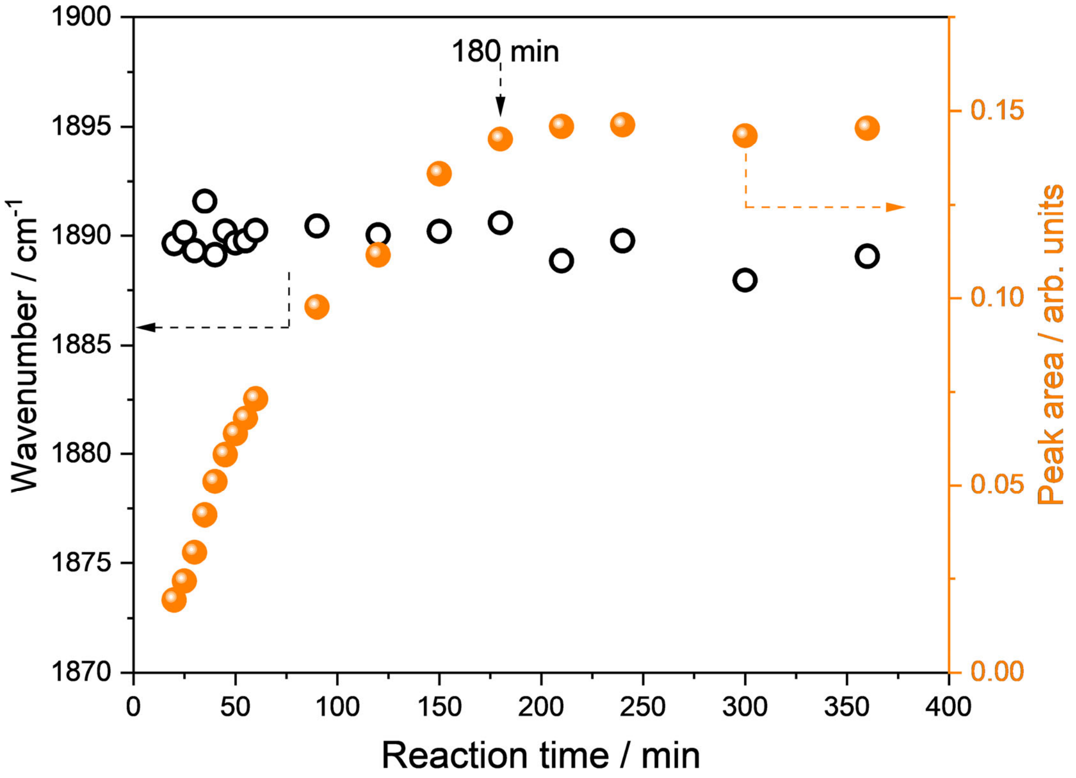

Supplementary Fig. 11. Time-dependent peak position and peak area of CO adsorption on the Cu single sites of Cu/C_3N_4 SACs during CO_2RR process at −1.2 V.

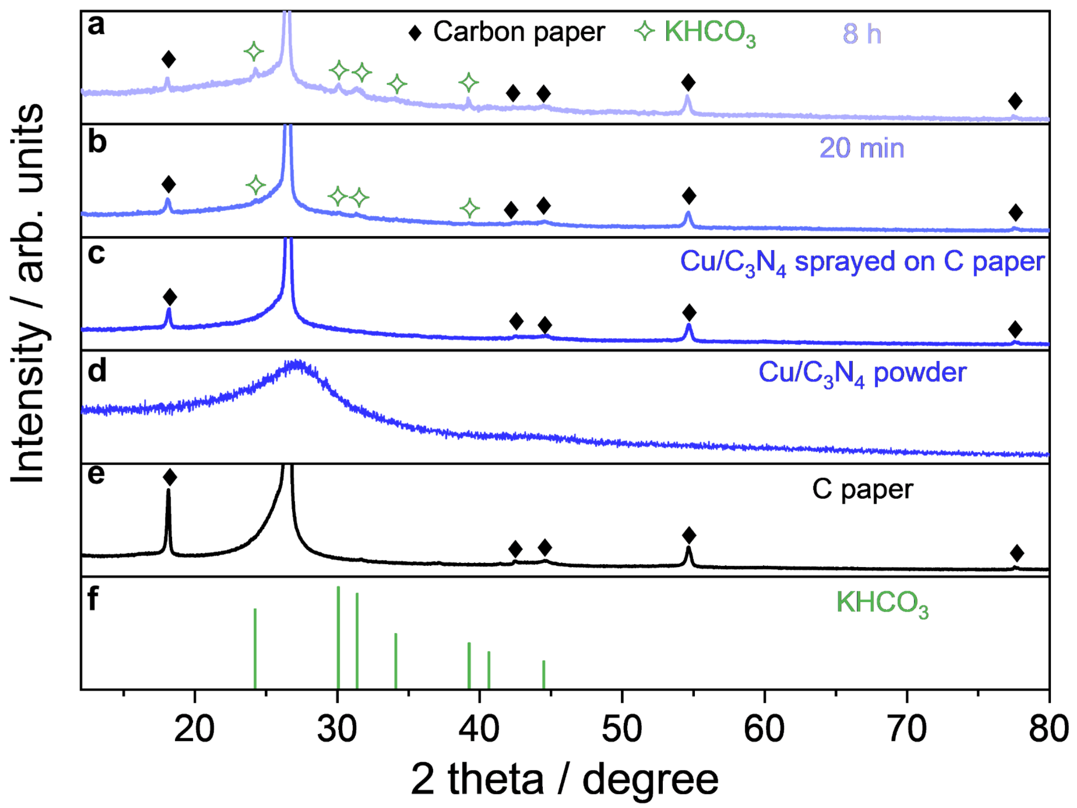

Supplementary Fig. 12. XRD patterns of carbon paper sprayed with Cu/C_3N_4 SACs after the CO_2RR at −1.2 V in 0.5 M CO_2-saturated $KHCO_3$ for (**a**) 8 h, (**b**) 20 min. (**c**) XRD pattern of carbon paper sprayed with Cu/C_3N_4 SACs before the CO_2RR. XRD patterns of (**d**) Cu/C_3N_4 powder and (**e**) carbon paper. (**f**) Standard XRD pattern of monoclinic $KHCO_3$ (JCPDS 01-070-0995). The XRD peak of Cu/C_3N_4 powder at 27.4º corresponds to the graphitic interlayer stacking.

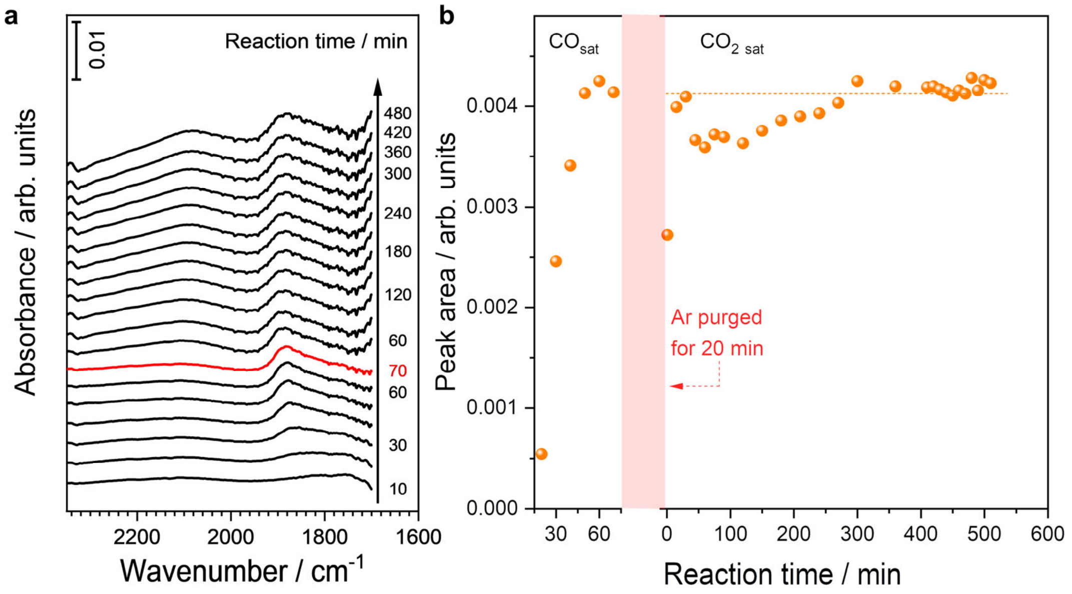

Supplementary Fig. 13. (**a**) Time-dependent SEIRA spectra of Cu/C_3N_4 SACs in CO/CO_2-saturated 0.5 M $KHCO_3$ solution (reference spectrum collected at the open circuit potential). CO was purged into 0.5 M $KHCO_3$ solution for the first 70 min. Then, Ar was purged into the electrolyte at OCP for 20 min to extract adsorbed CO. Subsequently, CO_2 was purged into 0.5 M $KHCO_3$ solution. (**b**) Time-dependent peak area of CO adsorption on Cu single sites. The peak area of CO adsorption on Cu single sites reaches a plateau until 50 min in CO-saturated 0.5 M $KHCO_3$ solution, indicating the slow mass transfer of CO to the Cu single sites.

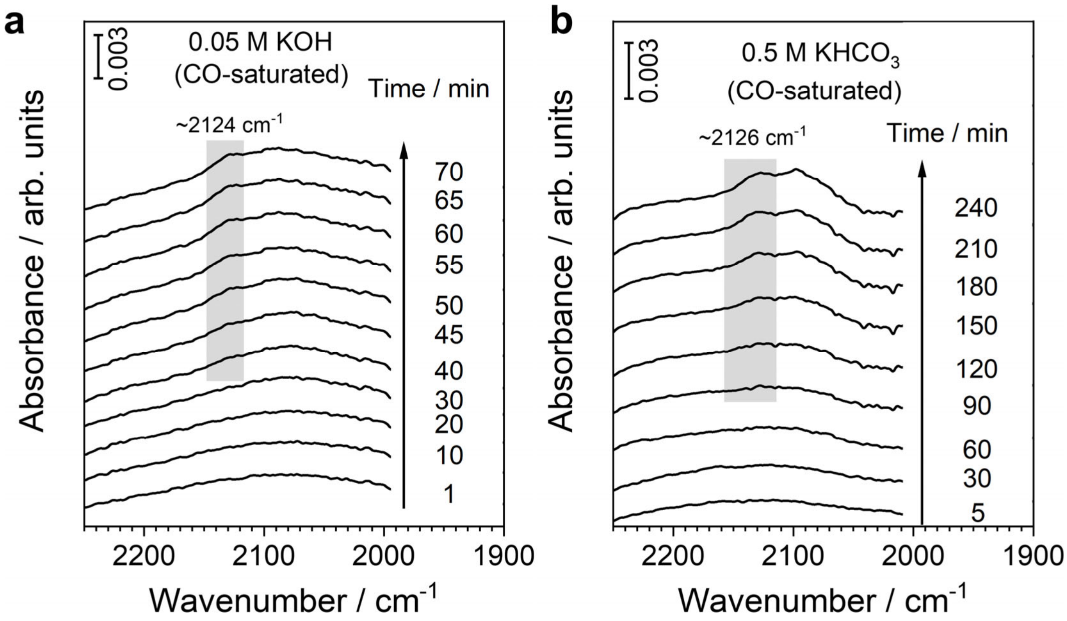

Supplementary Fig. 14. Time-dependent SEIRA spectra on the surfaces of Au films at −0.4 V vs RHE in (**a**) CO-saturated 0.05 M KOH solution and (**b**) CO-saturated 0.5 M $KHCO_3$ solution.

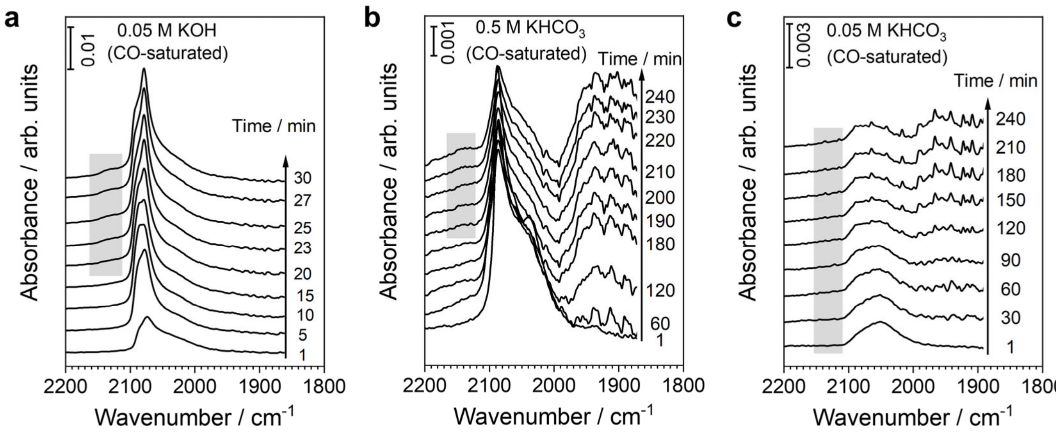

Supplementary Fig. 15. Time-dependent SEIRA spectra on the surfaces of Cu films at −0.4 V vs RHE in (**a**) CO-saturated 0.05 M $KHCO_3$ solution, (**b**) CO-saturated 0.5 M $KHCO_3$ solution, and (**c**) CO-saturated 0.05 KOH solution.

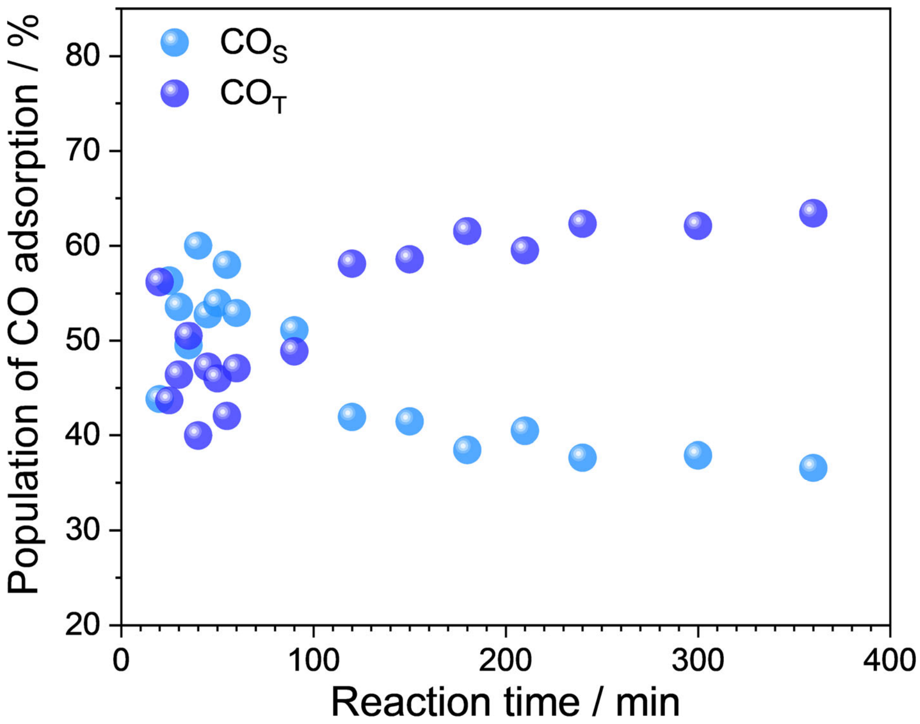

Supplementary Fig. 16. Time-dependent population of CO adsorptions on metallic Cu sites during the CO_2RR in CO_2-saturated 0.5 M $KHCO_3$ solution at −1.2 V from *operando* SEIRA spectra.

4. Procedure for the Quantification of Evolution Rate

The formation of metallic Cu sites can be described as the following elementary reaction:

$$Cu^{\delta+} + \delta e^{-} \rightarrow Cu_0 \qquad \text{(Eq. S1)}$$

The conversion of Cu single sites to metallic Cu sites only involves electron transfer process. Therefore, the kinetics involved could be expressed as a first-order reaction:

$$\text{rate} = k\left[Cu^{\delta+}\right] = \frac{d[Cu_0]}{dt} \qquad \text{(Eq. S2)}$$

where k is the rate constant, $[Cu^{\delta+}]$ and $[Cu_0]$ represents the concentrations of Cu single sites and metallic Cu sites in the catalysts, respectively. In the present study, because the reactant (Cu single sites) is in great excess relative to the product (metallic Cu sites), the concentration of Cu single sites remains at a constant level during the evolution process. The density of adsorbed CO molecules on the surfaces of 2-nm Cu nanoparticles is set as a constant. Therefore, the rate constant k can be extracted using the following equation:

$$k = \frac{d\frac{[Cu_0]}{[Cu^{\delta+}]}}{dt} \qquad \text{(Eq. S3)}$$

$[Cu^{\delta+}]$ and $[Cu_0]$ are determined based on the peak area of CO adsorptions on Cu single sites and metallic Cu sites, respectively. Since the concentration of Cu single sites remains at a constant level during the evolution process, CO adsorption on Cu singles sites is utilized as an internal standard for the quantification of $[Cu_0]$.

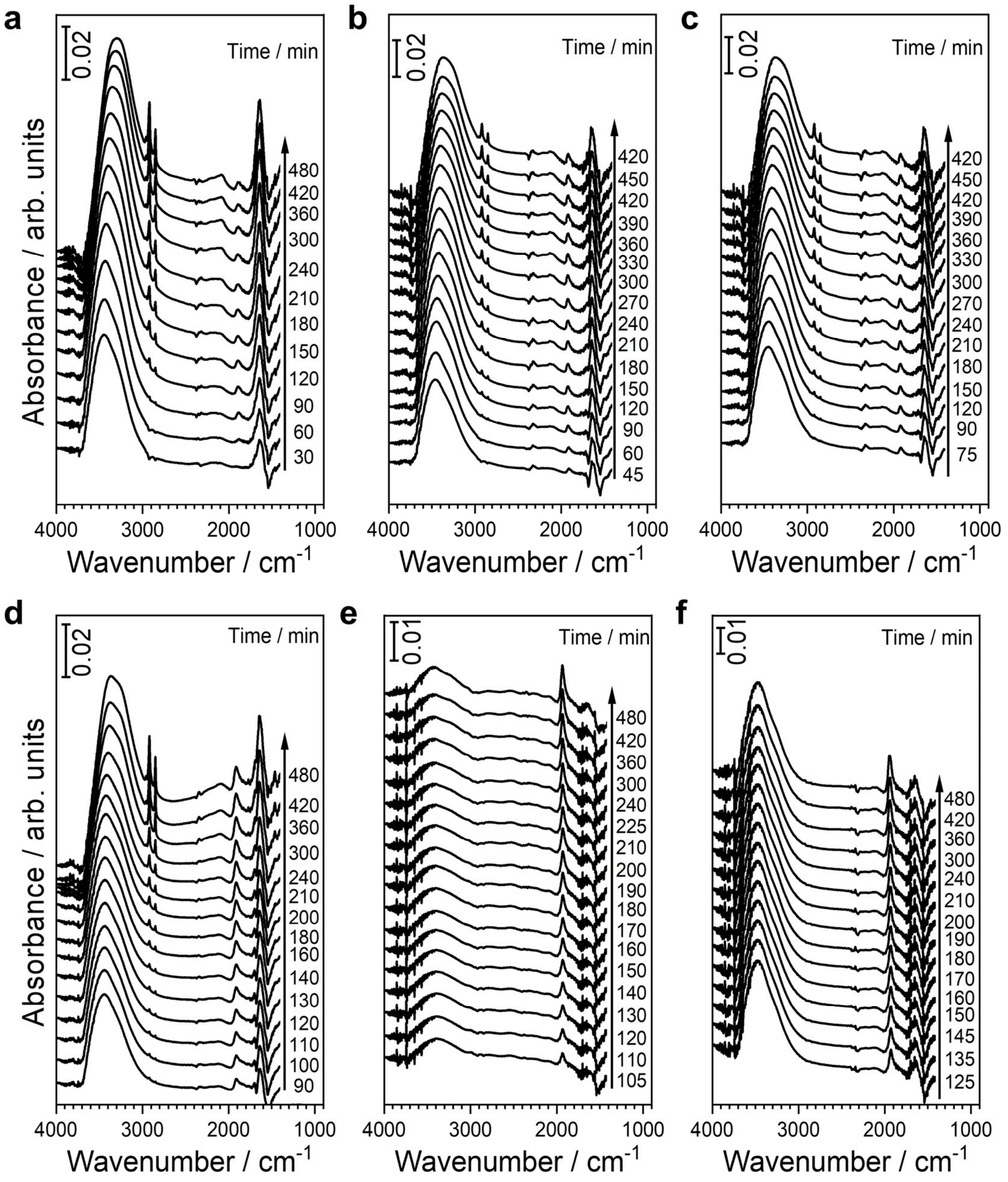

Supplementary Fig. 17. Time-dependent SEIRA spectra of the Cu/C_3N_4 SACs collected in CO_2-saturated 0.5 M $KHCO_3$ solution at a potential of (**a**) −1.1 V, (**b**) −1.0 V, (**c**) −0.9 V, (**d**) −0.8 V, (**e**) −0.7 V, and (**f**) −0.6 V.

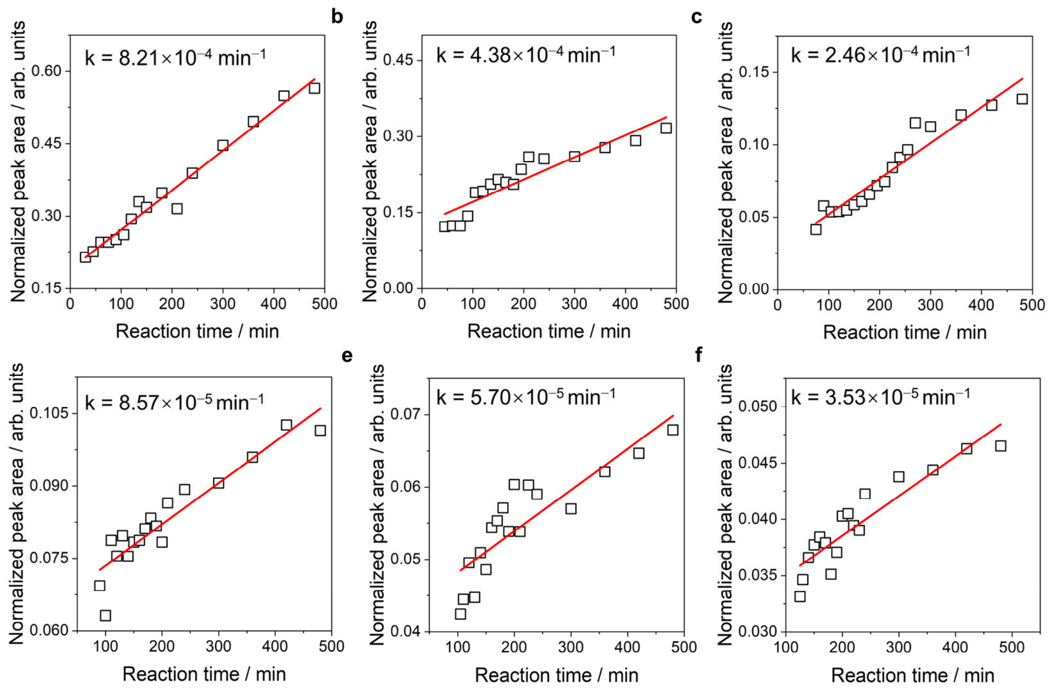

Supplementary Fig. 18. The peak area of CO adsorptions on metallic Cu sites of the Cu/C_3N_4 SACs from *operando* SEIRA spectra and the corresponding evolution rate constant at a potential of (**a**) −1.1 V, (**b**) −1.0 V, (**c**) −0.9 V, (**d**) −0.8 V, (**e**) −0.7 V, and (**f**) −0.6 V.

5. Morphology and Structure Characterizations of the Cu/C_3N_4 SACs post CO_2RR

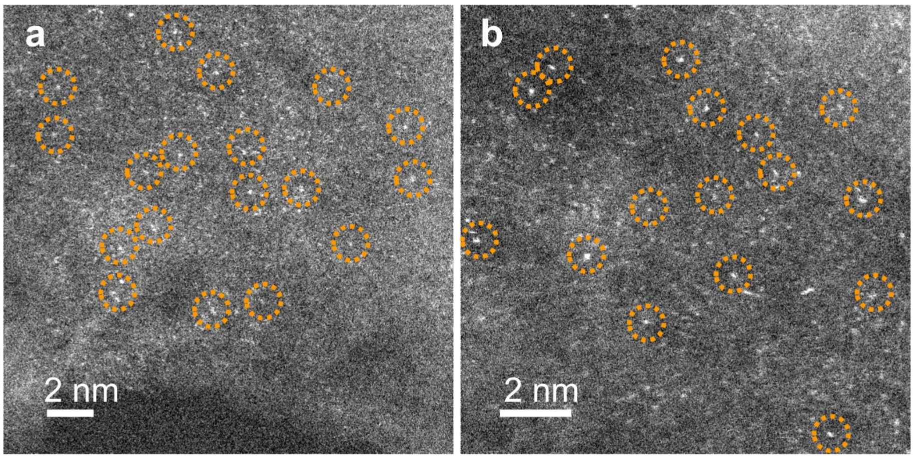

Supplementary Fig. 19. HAADF-STEM images of the Cu/C_3N_4 SACs post CO_2RR at −1.2 V for 8 h (**a** and **b**). There is decent amount of Cu single sites after the CO_2RR for 8 h, indicating that the evolution of Cu single atoms to Cu nanoparticles is minor.

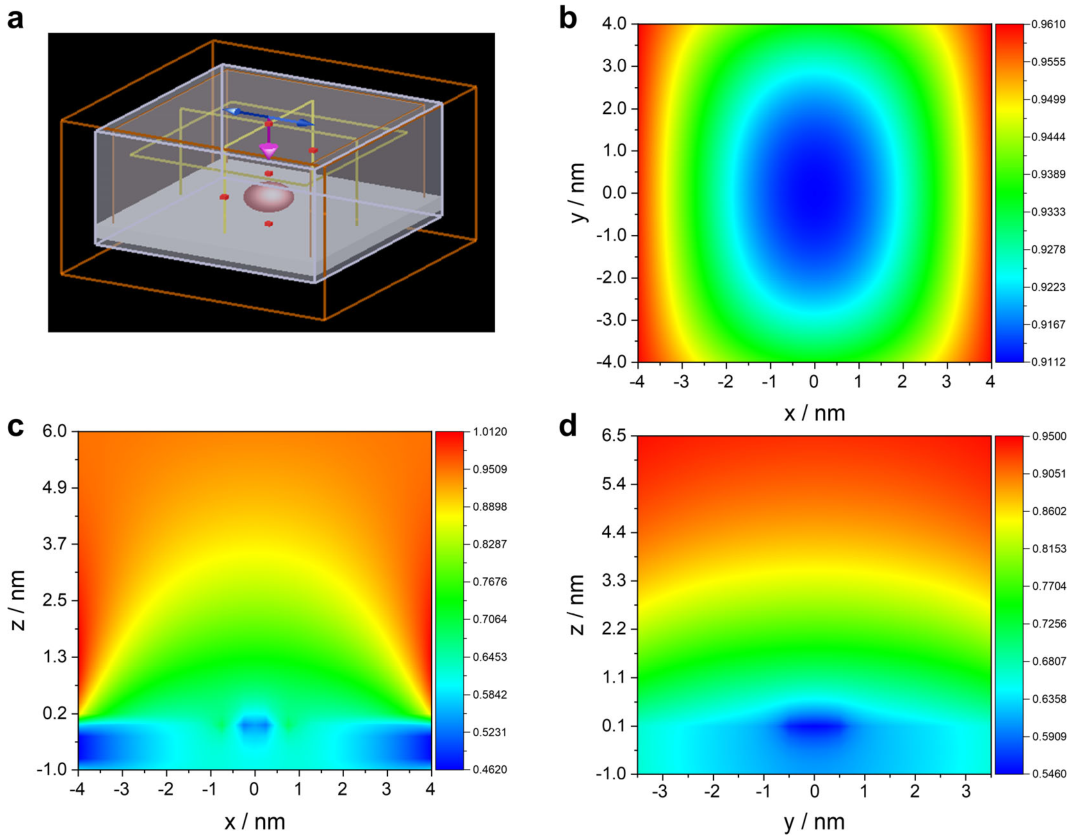

Supplementary Fig. 20. The finite difference time domain (FDTD) simulations of the local electromagnetic field for 2-nm Cu nanoparticles. (**a**) 3D model of 2-nm Cu nanoparticles. Simulated near-field enhancement $|E|^2$ of for 2-nm Cu nanoparticles on (**b**) xy plane, (**c**) xz plane and (**d**) yz plane.

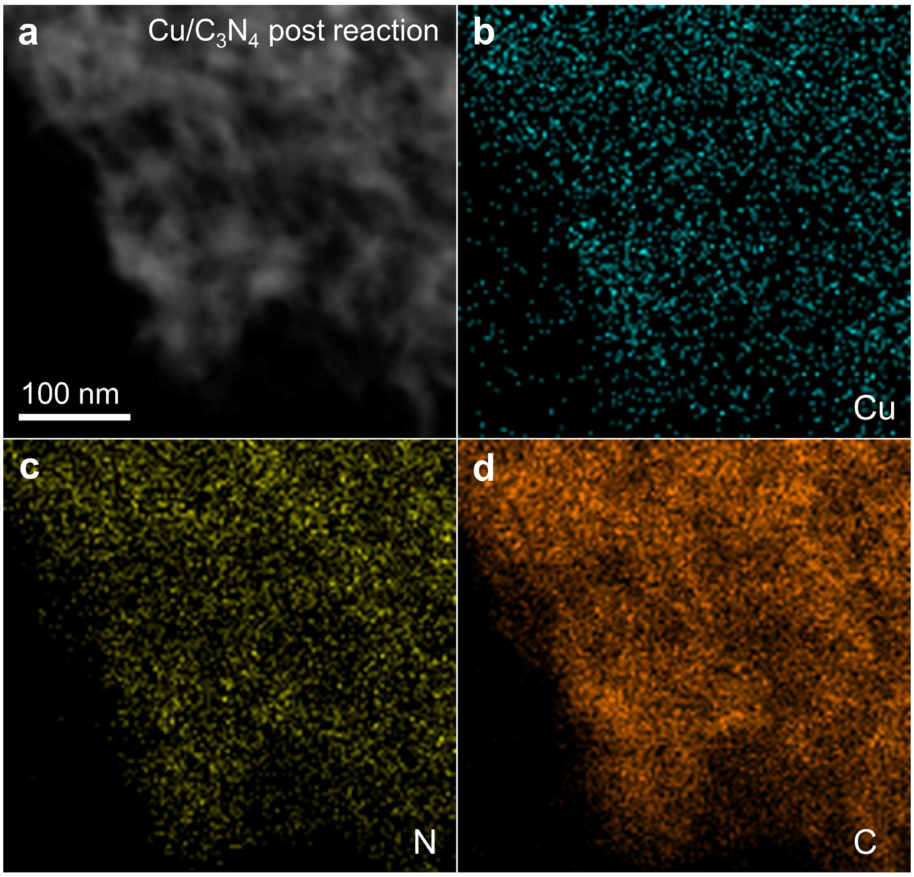

Supplementary Fig. 21. (**a**) HAADF-STEM images of the Cu/C_3N_4 SACs post CO_2RR at −1.2 V for 8 h. **b−d** The corresponding EDX mapping of elemental distributions for (**b**) Cu, (**c**) N, and (**d**) C.

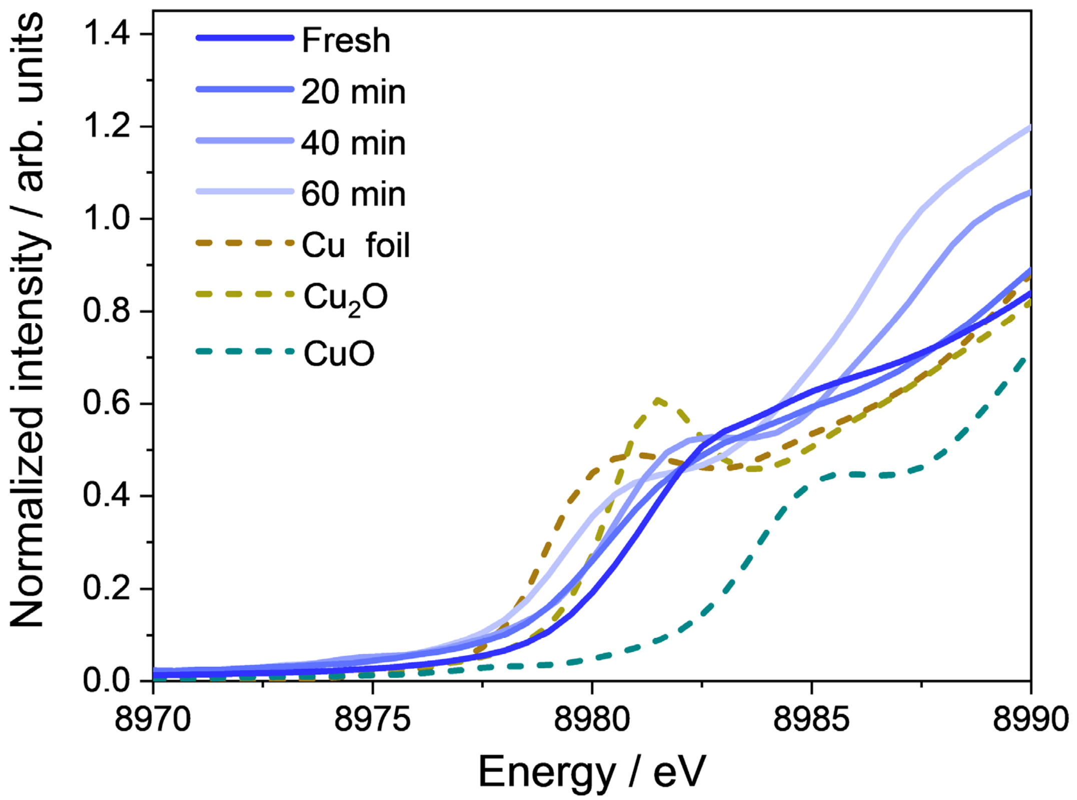

Supplementary Fig. 22. Time-dependent *in situ* XAFS spectra of the Cu/C_3N_4 SACs during the CO_2RR in CO_2-saturated 0.5 M $KHCO_3$ solution at −1.2 V.

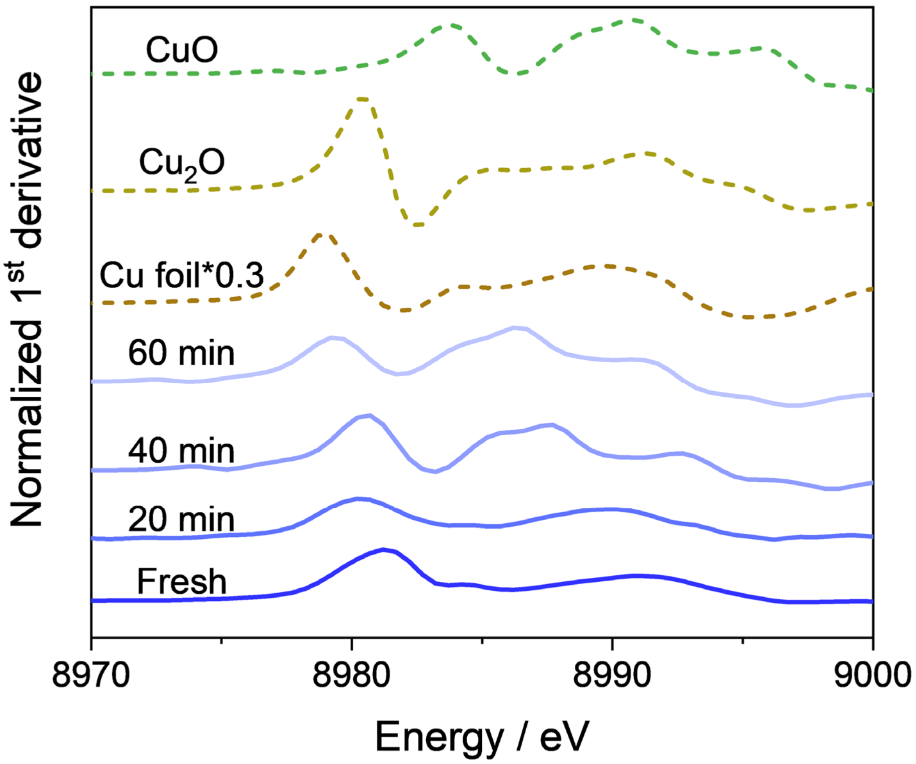

Supplementary Fig. 23. Time-dependent first derivative spectra of normalized Cu K-edge XANES of the Cu/C_3N_4 SACs during the CO_2RR in CO_2-saturated 0.5 M $KHCO_3$ solution at −1.2 V.

6. Morphology and Structure Characterizations of the CuPc, Cu-NC, and Cu-SNC Catalysts

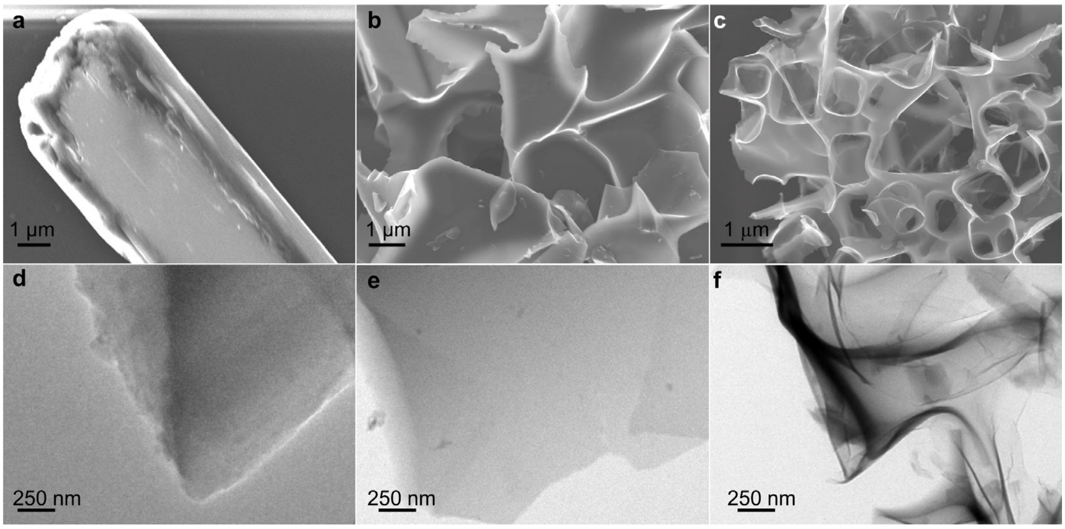

Supplementary Fig. 24. SEM images of (**a**) CuPc, (**b**) Cu-NC, and (**c**) Cu-SNC catalysts. TEM images of (**d**) CuPc, (**e**) Cu-NC, and (**f**) Cu-SNC catalysts.

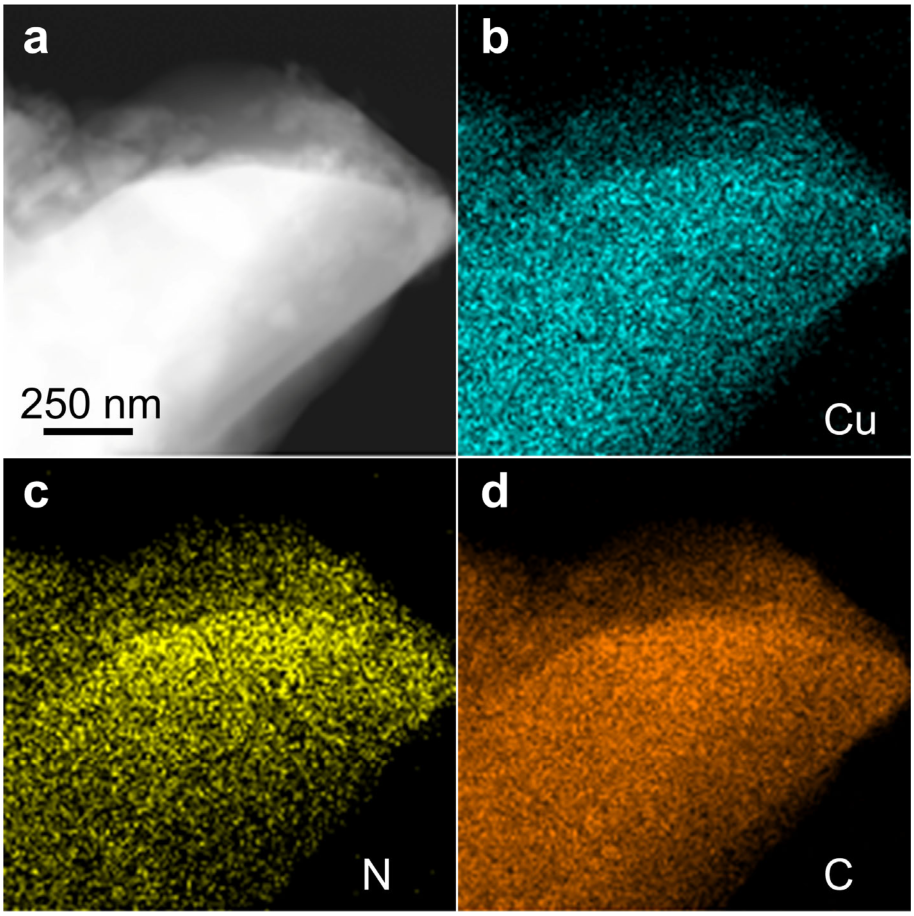

Supplementary Fig. 25. (**a**) HAADF-STEM images of the CuPc SACs. **b−d** The corresponding EDX mapping of elemental distributions for (**b**) Cu, (**c**) N, and (**d**) C.

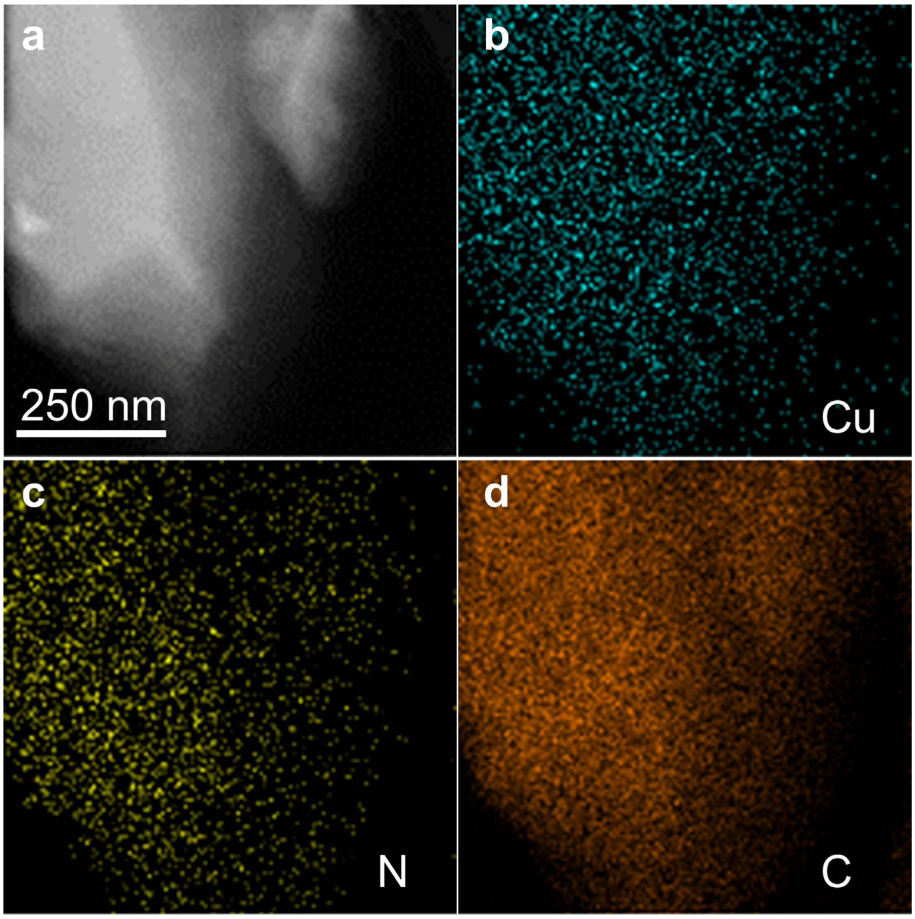

Supplementary Fig. 26. (**a**) HAADF-STEM images of the Cu-NC SACs. **b−d** The corresponding EDX mapping of elemental distributions for (**b**) Cu, (**c**) N, and (**d**) C.

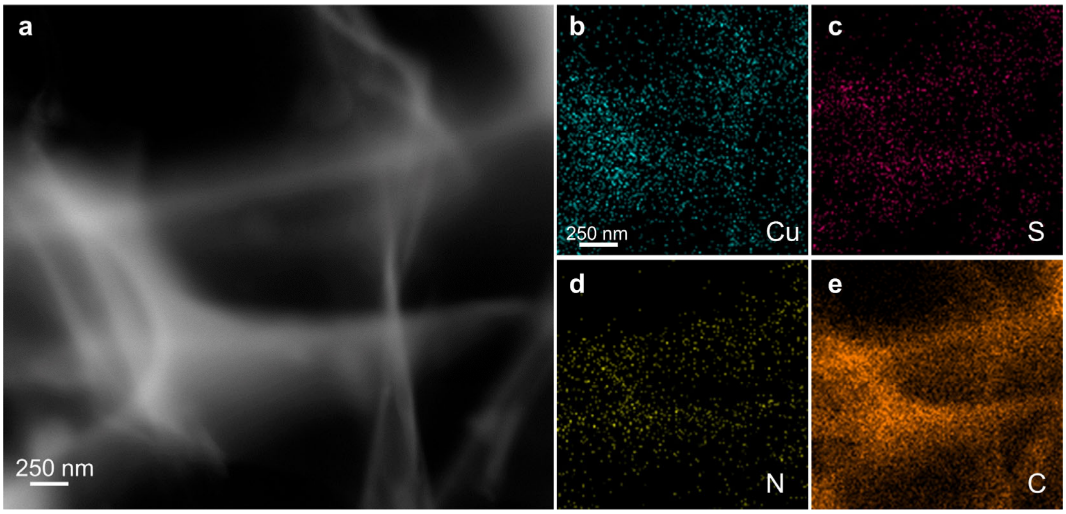

Supplementary Fig. 27. (**a**) HAADF-STEM images of the Cu-SNC SACs. **b−e** The corresponding EDX mapping of elemental distributions for (**b**) Cu, (**c**) S, (**d**) N, and (**e**) C.

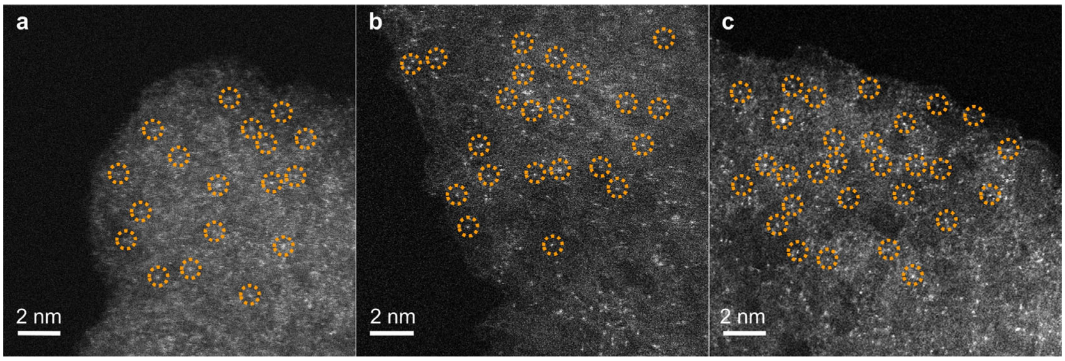

Supplementary Fig. 28. HAADF-STEM images of (**a**) CuPc, (**b**) Cu-NC, and (**c**) Cu-SNC SACs (selected single Cu atoms are marked by the orange circles).

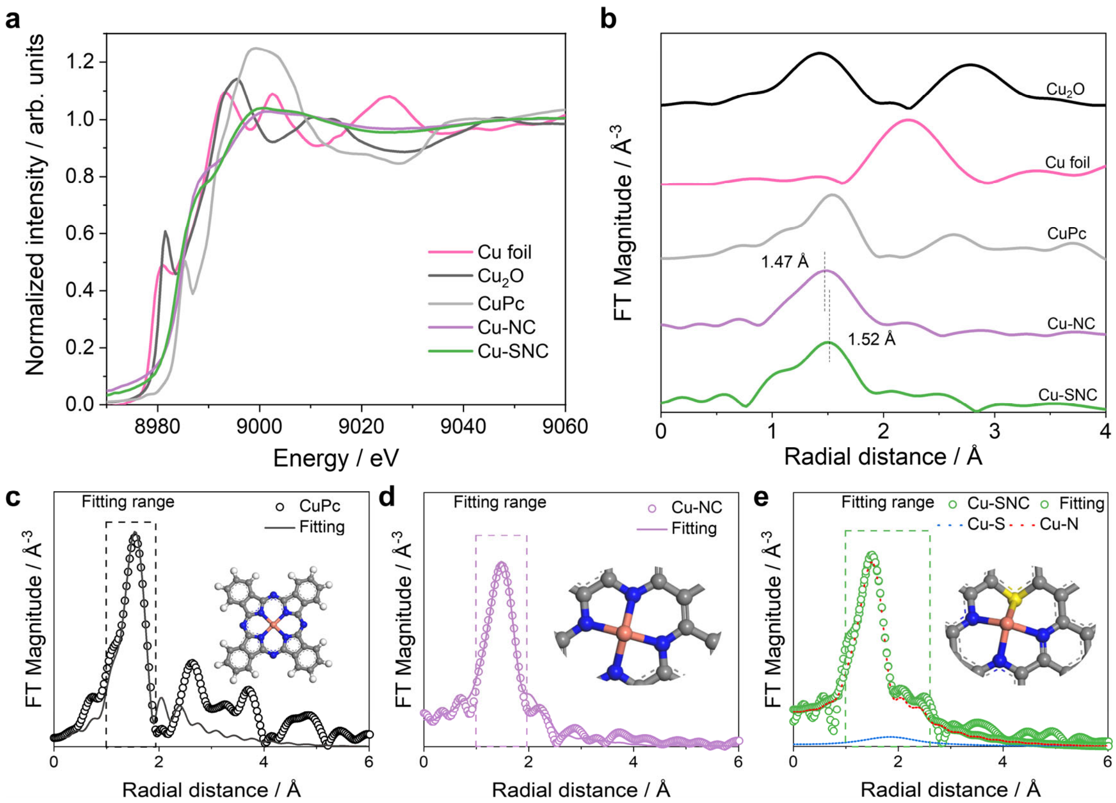

Supplementary Fig. 29. (**a**) Normalized XANES spectra at the Cu K-edge of CuPc, Cu-NC, and Cu-SNC SACs. (**b**) FT-EXAFS spectra of Cu_2O, Cu foil, CuPc, Cu-NC, and Cu-SNC SACs. Note that the intensity of the EXAFS spectrum of Cu foil is amplified by 0.3 times. **c−e** Cu K-edge EXAFS fitting curves for (**c**) CuPc, (**d**) Cu-NC, and (**e**) Cu-SNC SACs in the range of 1.0−1.9 Å, 1.0−1.9 Å, and 1.0−2.6 Å, respectively. The k^2-weighted EXAFS spectra at Cu K-edge for the CuPc, Cu-NC and Cu-SNC catalysts show a peak located at 1.50 Å, which is attributed to the main scattering feature of Cu-N/O interaction. There is a shift of 0.05 Å for the main scattering peak between the Cu-NC and Cu-SNC SACs, which is likely due to the interaction between Cu and S atoms.

7. *Operando* SEIRAS Measurements of the CuPc, Cu-NC, and Cu-SNC Catalysts

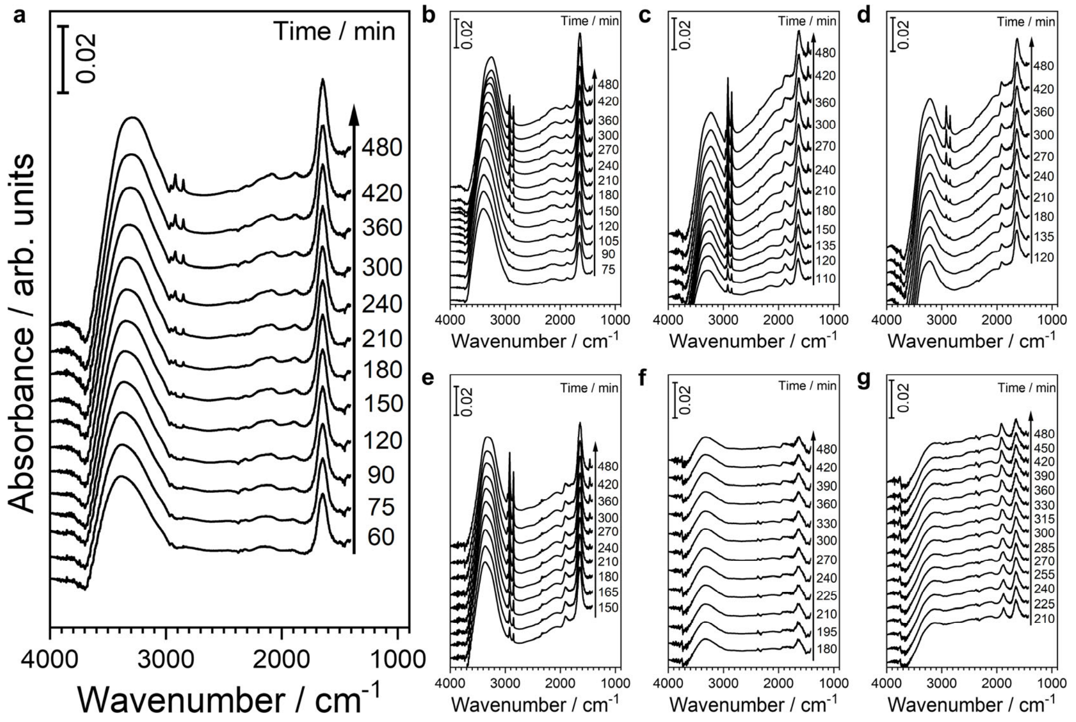

Supplementary Fig. 30. Time-dependent SEIRA spectra of the CuPc SACs collected in CO_2-saturated 0.5 M $KHCO_3$ solution at a potential of (**a**) −1.2 V, (**b**) −1.1 V, (**c**) −1.0 V, (**d**) −0.9 V, (**e**) −0.8 V, (**f**) −0.7 V, and (**g**) −0.6 V.

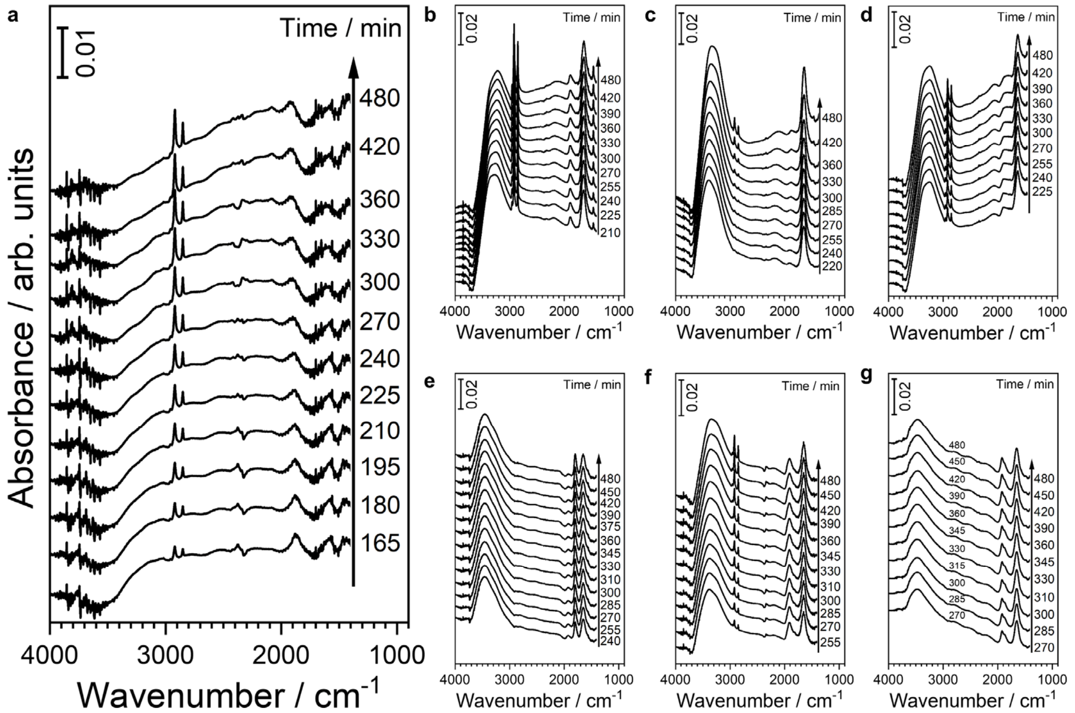

Supplementary Fig. 31. Time-dependent SEIRA spectra of the Cu-NC SACs collected in CO_2-saturated 0.5 M $KHCO_3$ solution at a potential of (**a**) −1.2 V, (**b**) −1.1 V, (**c**) −1.0 V, (**d**) −0.9 V, (**e**) −0.8 V, (**f**) −0.7 V, and (**g**) −0.6 V.

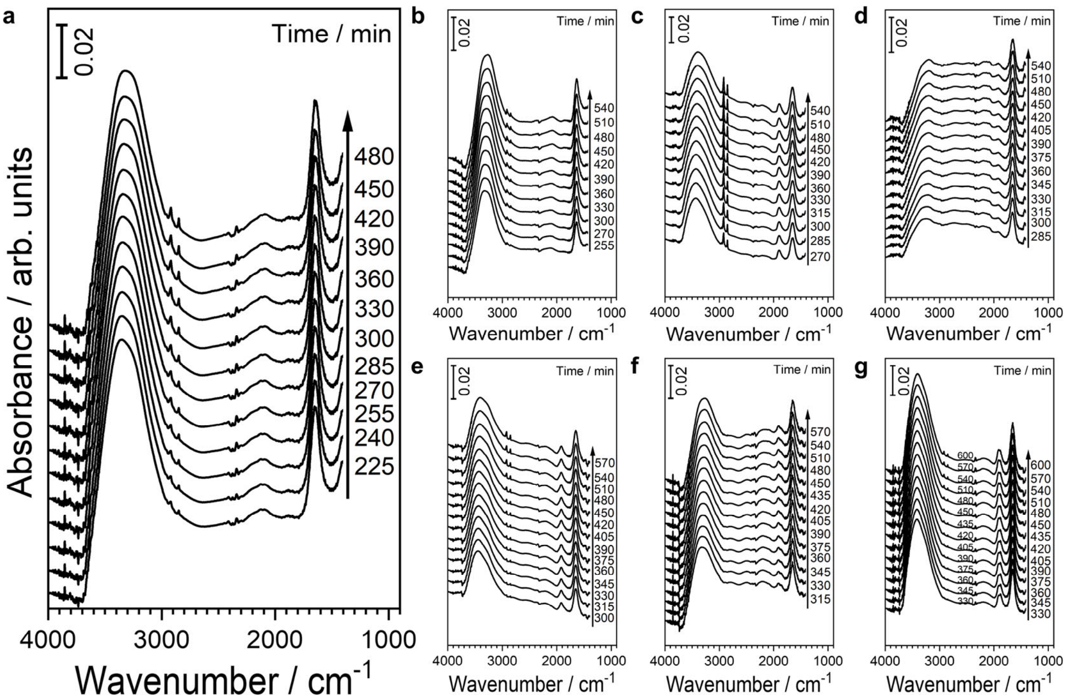

Supplementary Fig. 32. Time-dependent SEIRA spectra of the Cu-SNC SACs collected in CO_2-saturated 0.5 M $KHCO_3$ solution at a potential of (**a**) −1.2 V, (**b**) −1.1 V, (**c**) −1.0 V, (**d**) −0.9 V, (**e**) −0.8 V, (**f**) −0.7 V, and (**g**) −0.6 V.

8. Morphology Characterizations of the CuPc, Cu-NC, and Cu-SNC Catalysts Post CO_2RR

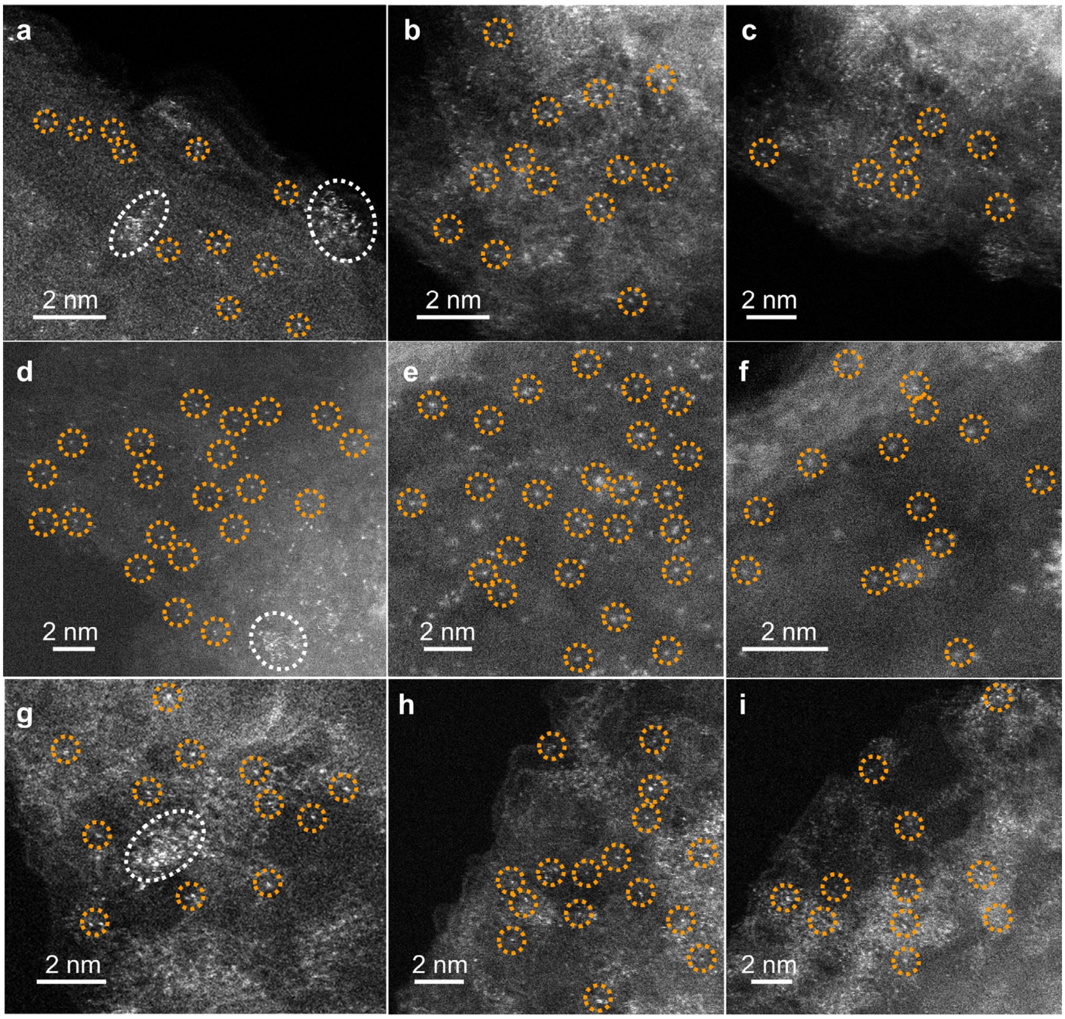

Supplementary Fig. 33. HAADF-STEM images of (**a**−**c**) CuPc, (**d**−**f**) Cu-NC and (**g**−**i**) Cu-SNC SACs post CO_2RR at −1.2 V for 8 h (selected single Cu atoms and nanoparticles are marked by the orange circles and white ellipses, respectively).

9. Evolution Rates of the CuPc, Cu-NC, and Cu-SNC Catalysts

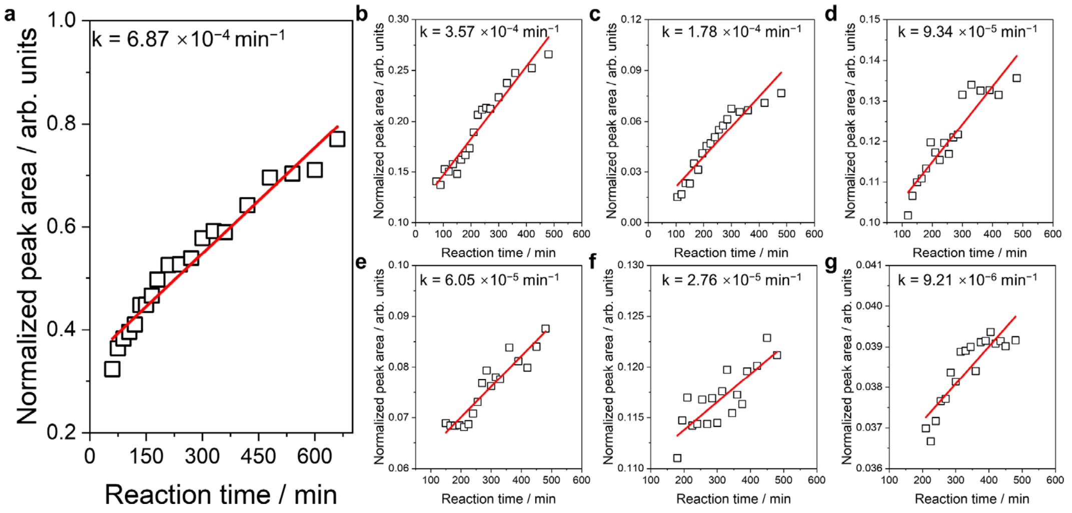

Supplementary Fig. 34. The peak area of CO adsorptions on metallic Cu sites of the CuPc SACs from *operando* SEIRA spectra and the corresponding evolution rate constant at a potential of (**a**) −1.2 V, (**b**) −1.1 V, (**c**) −1.0 V, (**d**) −0.9 V, (**e**) −0.8 V, (**f**) −0.7 V, and (**g**) −0.6 V.

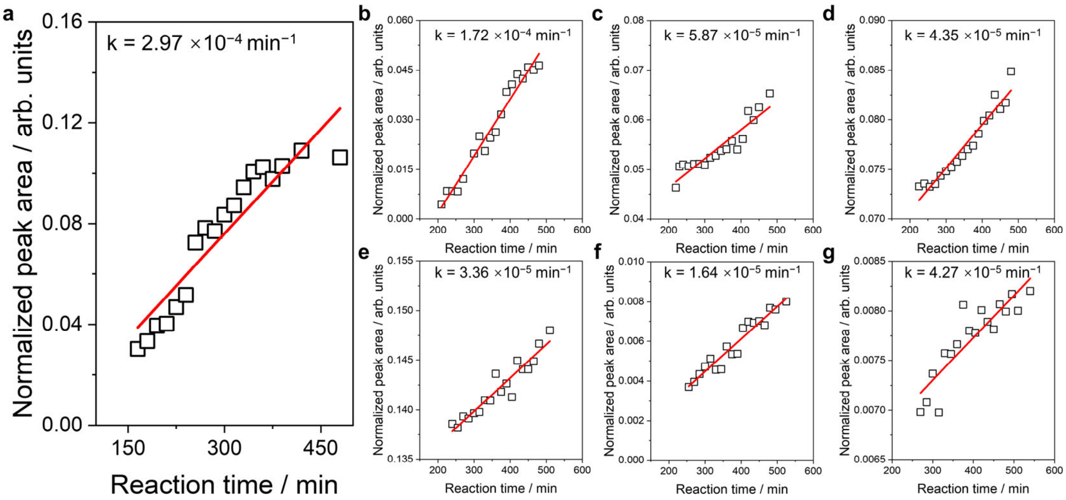

Supplementary Fig. 35. The peak area of CO adsorptions on metallic Cu sites of the Cu-NC SACs from *operando* SEIRA spectra and the corresponding evolution rate constant at a potential of (**a**) −1.2 V, (**b**) −1.1 V, (**c**) −1.0 V, (**d**) −0.9 V, (**e**) −0.8 V, (**f**) −0.7 V, and (**g**) −0.6 V.

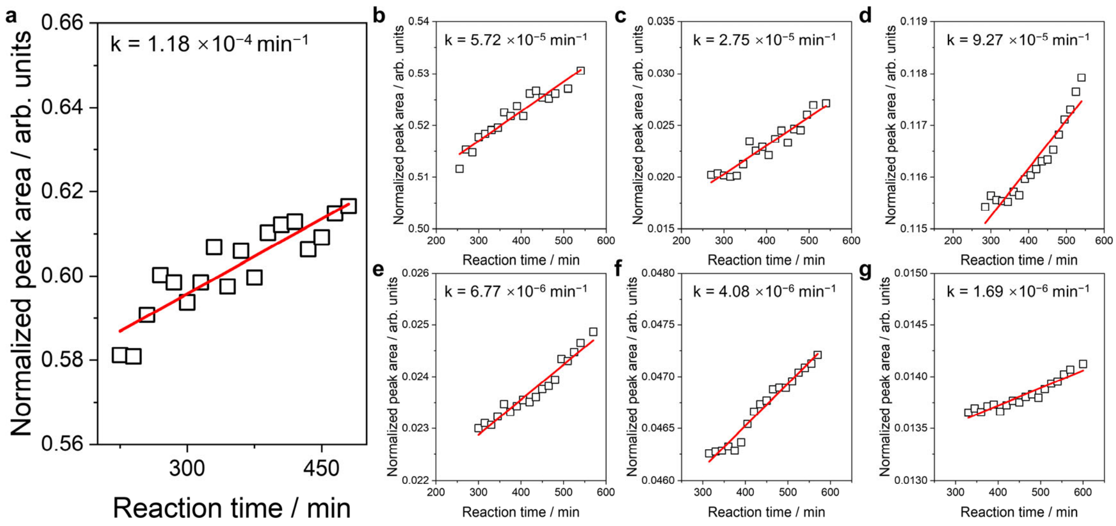

Supplementary Fig. 36. The peak area of CO adsorptions on metallic Cu sites of the Cu-SNC SACs from *operando* SEIRA spectra and the corresponding evolution rate constant at a potential of (**a**) −1.2 V, (**b**) −1.1 V, (**c**) −1.0 V, (**d**) −0.9 V, (**e**) −0.8 V, (**f**) −0.7 V, and (**g**) −0.6 V.

10. CO_2RR Performance of Cu SACs

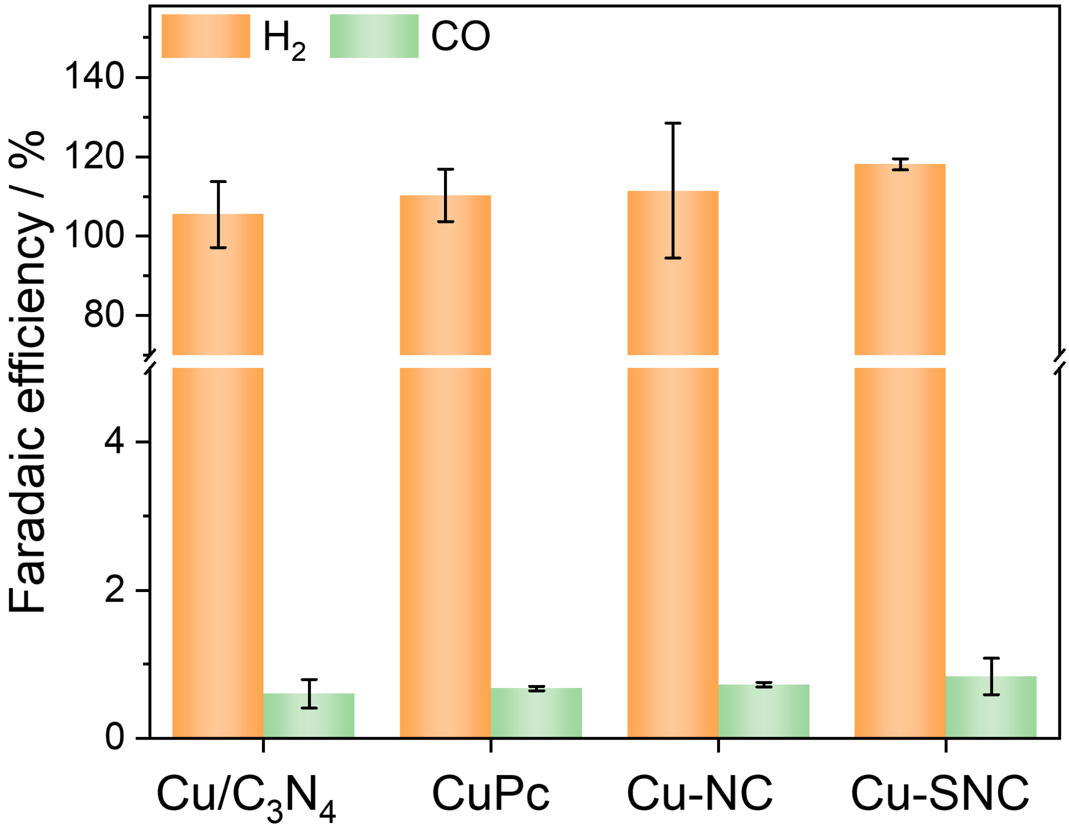

Supplementary Fig. 37. The CO_2RR performance of Cu/C_3N_4, CuPc, Cu-NC, and Cu-SNC SACs at −1.2 V during CO_2RR process for 15 min. The main products are H_2 and CO. The Faradaic efficiencies of CO are less than 1%. The Faradaic efficiency of CO follows the trend Cu-SNC > Cu-NC > CuPc > Cu/C_3N_4, with a Faradaic efficiency of CO reaching 0.83% on Cu-SNC SACs.

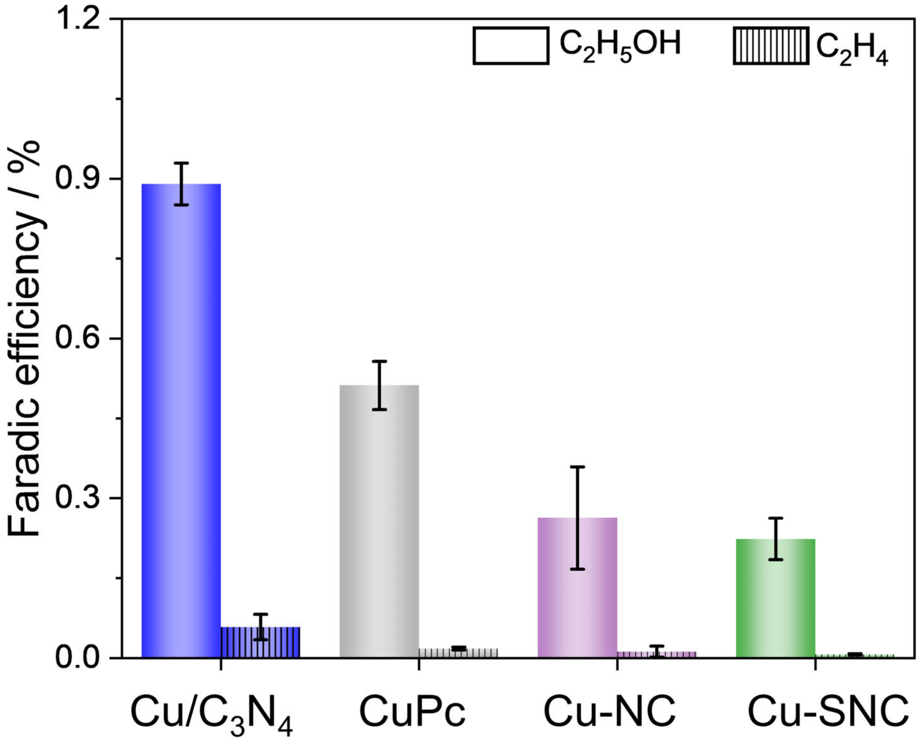

Supplementary Fig. 38. Faradaic efficiencies of C_2H_4 and C_2H_5OH on the Cu/C_3N_4, CuPc, Cu-NC, and Cu-SNC SACs during the CO_2RR at −1.2 V. The reaction time for Cu/C_3N_4, CuPc, Cu-NC, and Cu-SNC SACs is 30, 60, 200, and 300 min, respectively. There is no C_{2+} products produced heretofore.

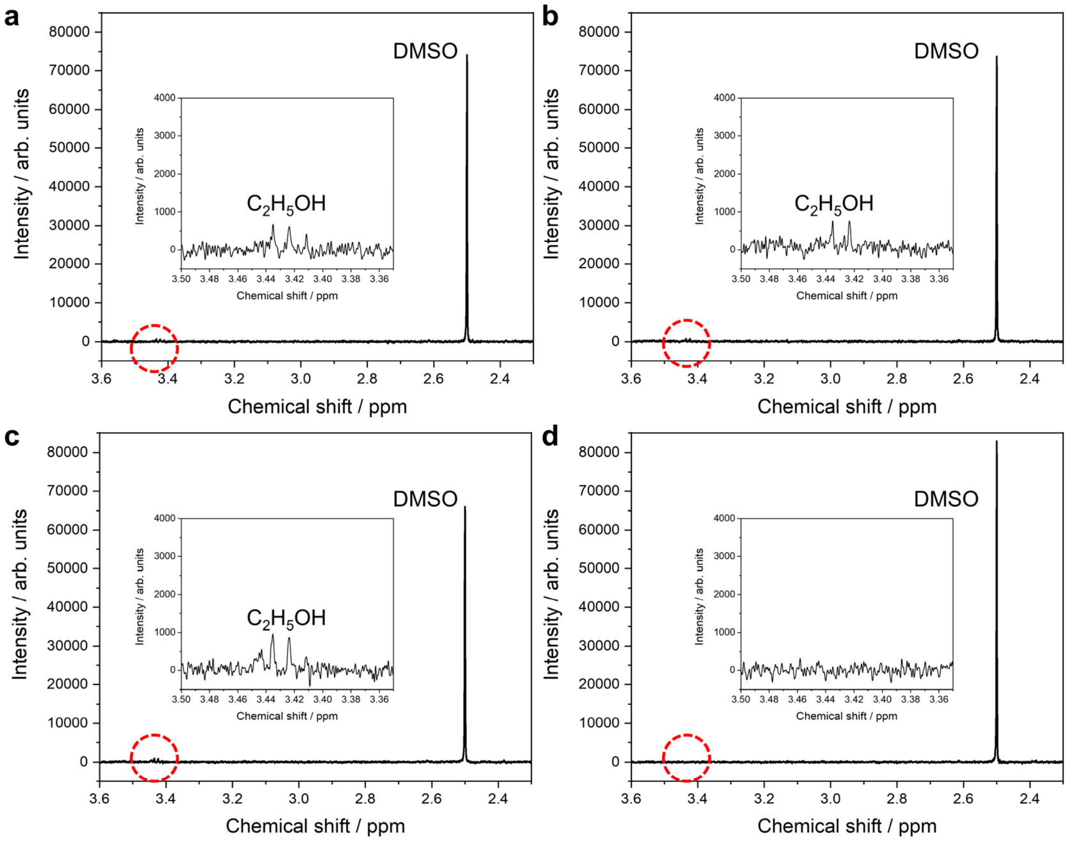

Supplementary Fig. 39. Typical ^{1}H NMR spectra of C_{2+} products with Cu/C_3N_4 SACs as the catalysts at −1.2 V vs RHE for (**a−c**) 30 min, (**d**) 15 min. **a**, **b**, and **c** show the ^{1}H NMR spectra in three parallel experiments.

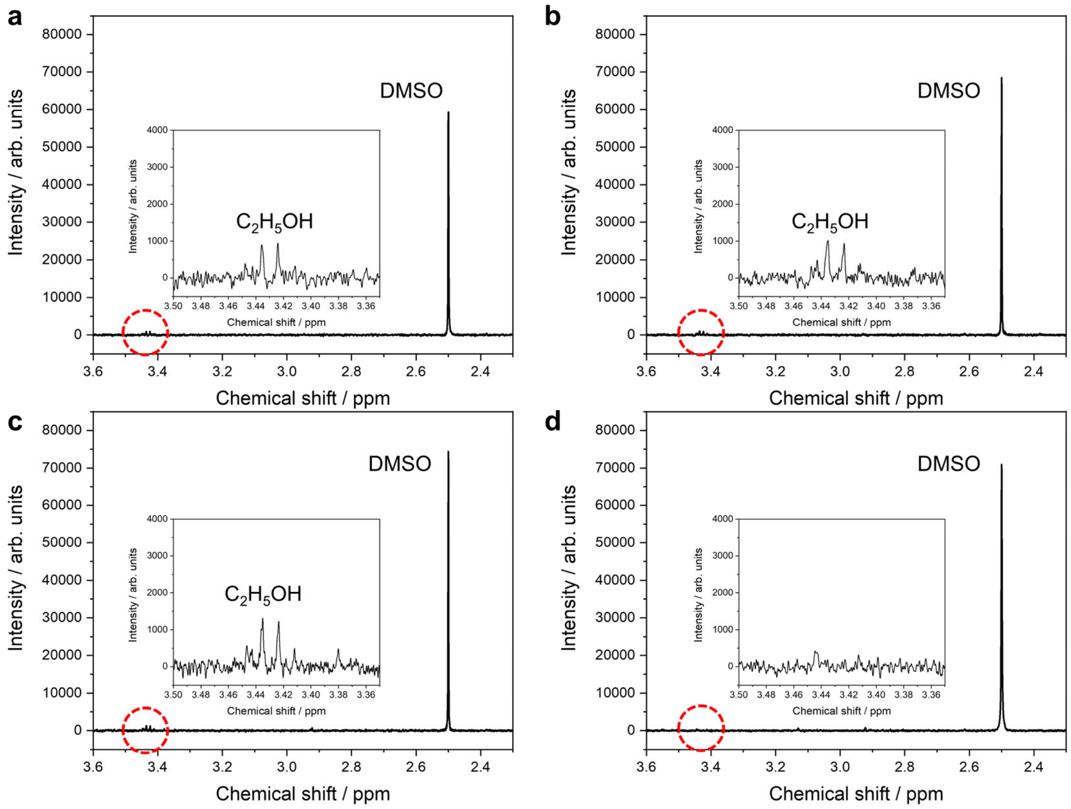

Supplementary Fig. 40. Typical ^{1}H NMR spectra of C_{2+} products with CuPc SACs as the catalysts at −1.2 V vs RHE for (**a–c**) 60 min, (**d**) 40 min. **a**, **b**, and **c** show the ^{1}H NMR spectra in three parallel experiments.

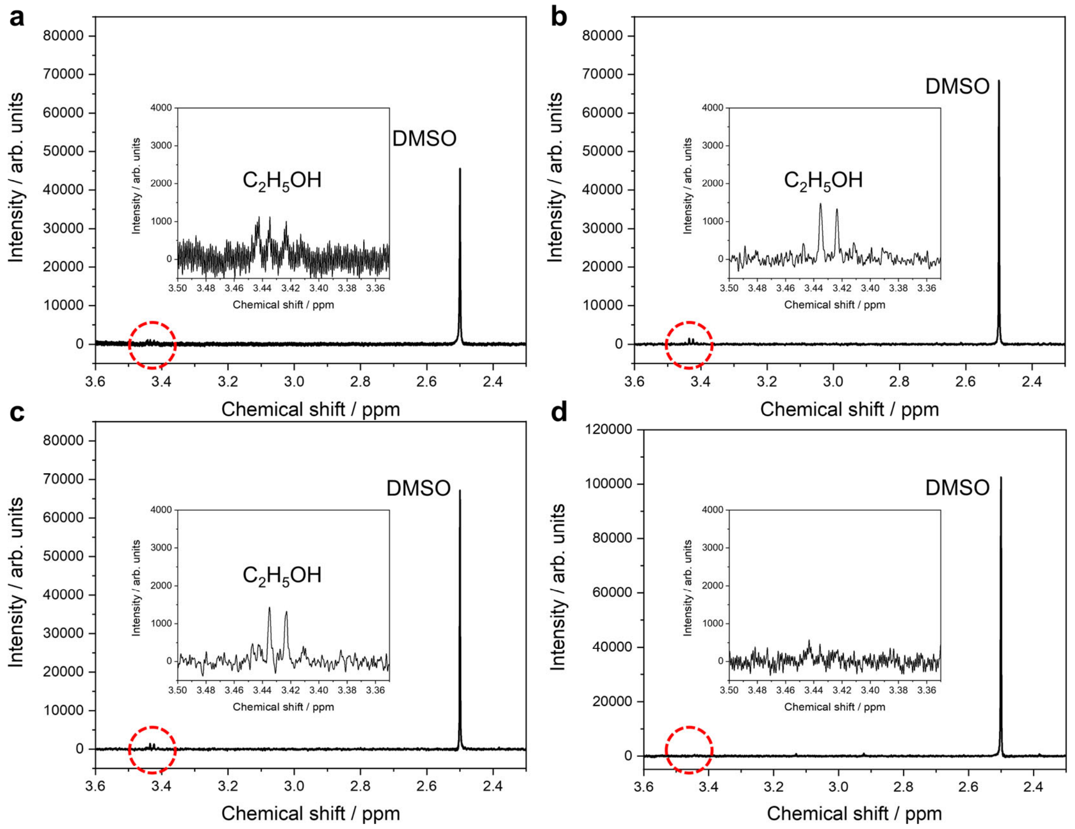

Supplementary Fig. 41. Typical ^{1}H NMR spectra of C_{2+} products with Cu-NC SACs as the catalysts at −1.2 V vs RHE for (**a–c**) 200 min, (**d**) 150 min. **a**, **b**, and **c** show the ^{1}H NMR spectra in three parallel experiments.

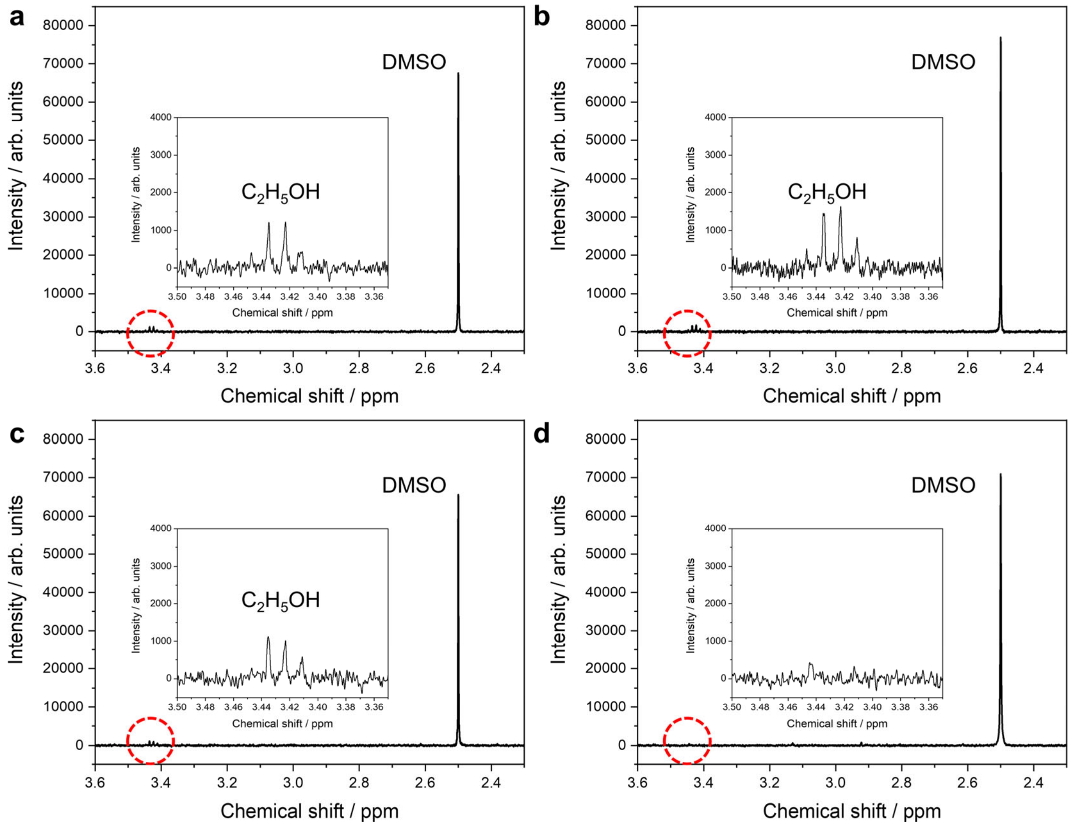

Supplementary Fig. 42. Typical ^{1}H NMR spectra of C_{2+} products with Cu-SNC SACs as the catalysts at −1.2 V vs RHE for (**a−c**) 300 min, (**d**) 240 min. **a**, **b**, and **c** show the ^{1}H NMR spectra in three parallel experiments.

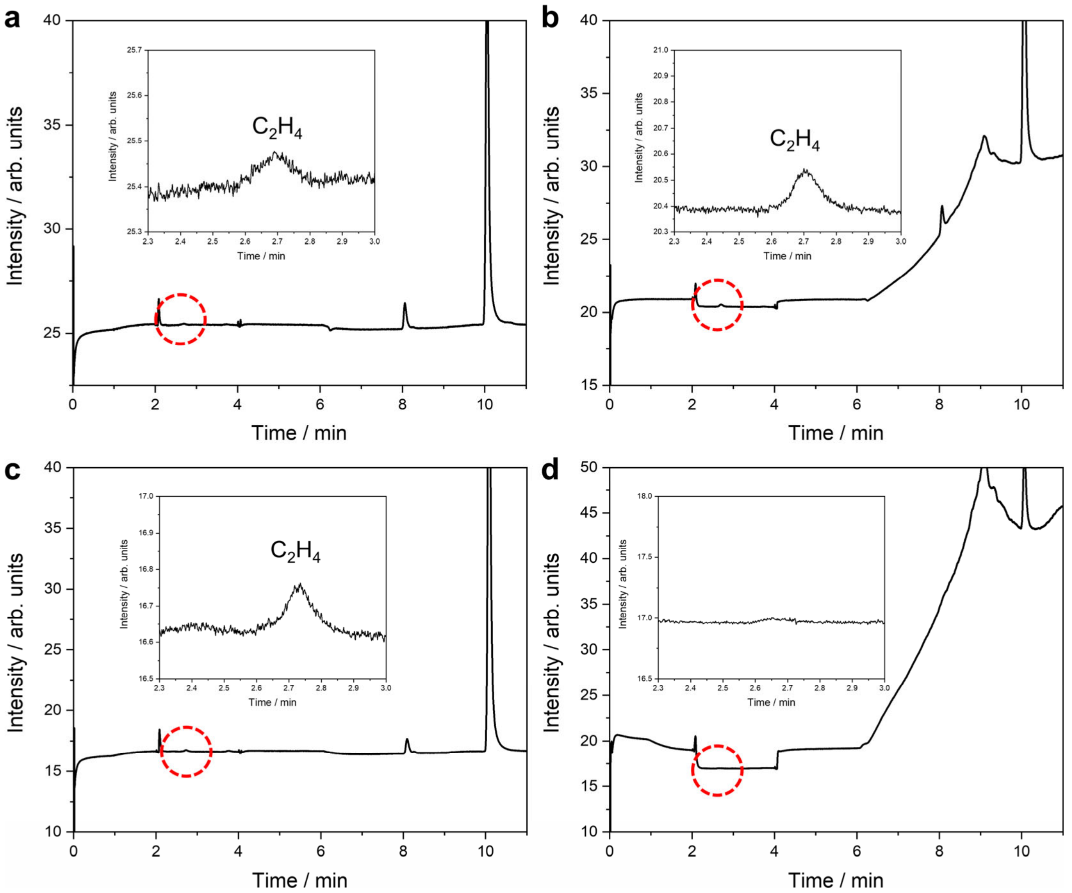

Supplementary Fig. 43. Typical GC traces of C_{2+} products with Cu/C_3N_4 SACs as the catalysts at −1.2 V vs RHE for (**a**–**c**) 30 min, (**d**) 15 min. **a**, **b**, and **c** show the GC traces in three parallel experiments.

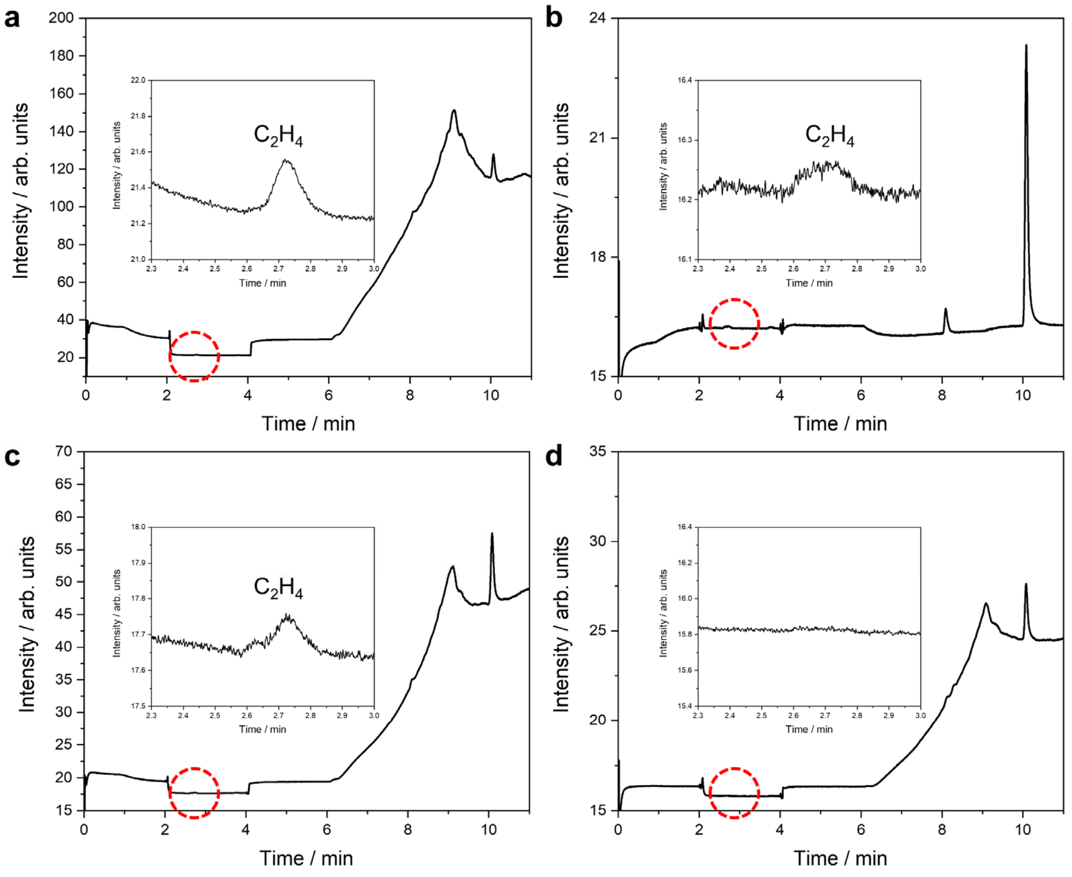

Supplementary Fig. 44. Typical GC traces of C_{2+} products with CuPc SACs as the catalysts at −1.2 V vs RHE for (**a**–**c**) 60 min, (**d**) 40 min. **a**, **b**, and **c** show the GC traces in three parallel experiments.

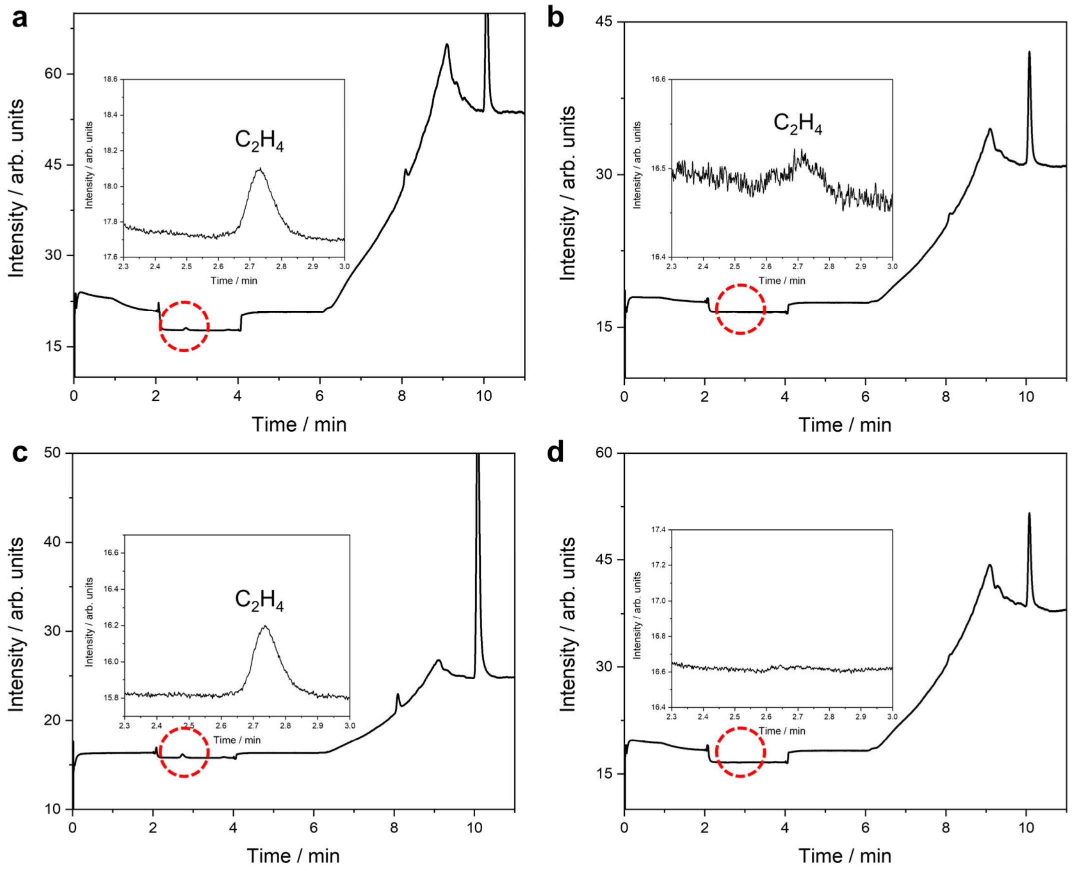

Supplementary Fig. 45. Typical GC traces of C_{2+} products with Cu-NC SACs as the catalysts at −1.2 V vs RHE for (**a**−**c**) 200 min, (**d**) 150 min. **a**, **b**, and **c** show the GC traces in three parallel experiments.

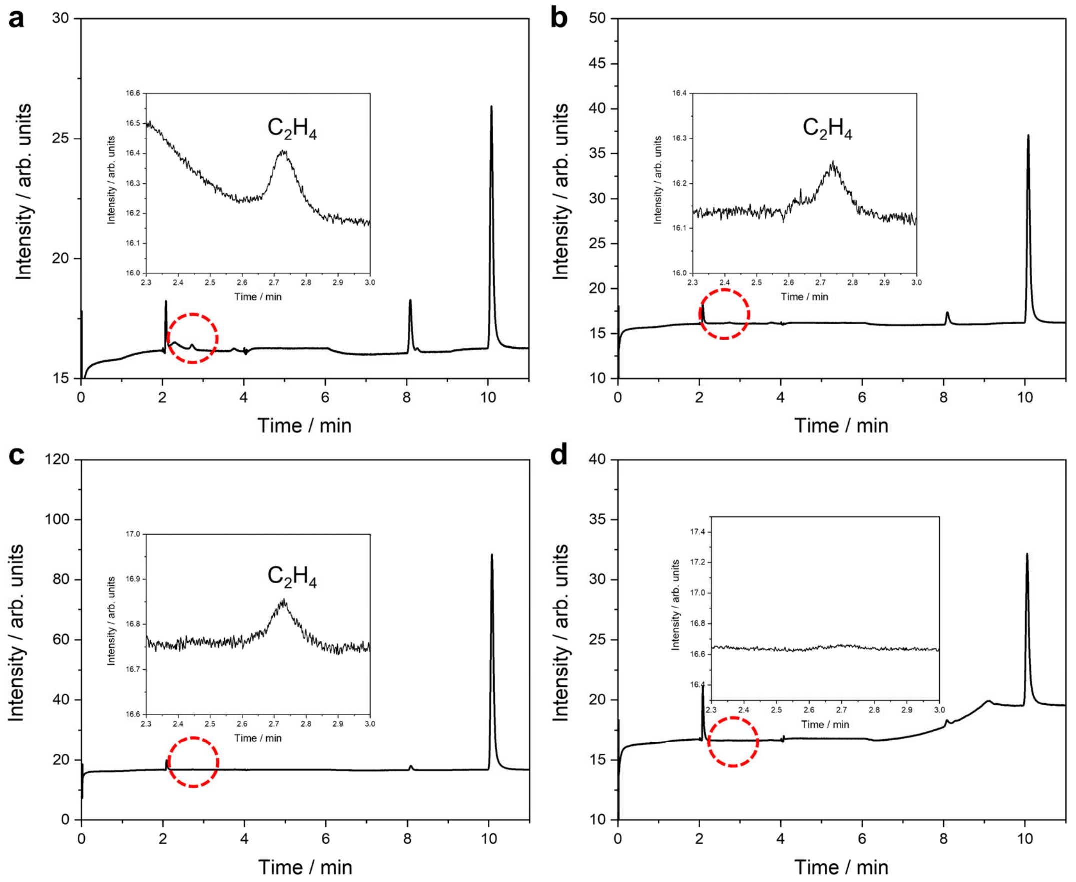

Supplementary Fig. 46. Typical GC traces of C_{2+} products with Cu-SNC SACs as the catalysts at −1.2 V vs RHE for (**a**−**c**) 300 min, (**d**) 240 min. **a**, **b**, and **c** show the GC traces in three parallel experiments.

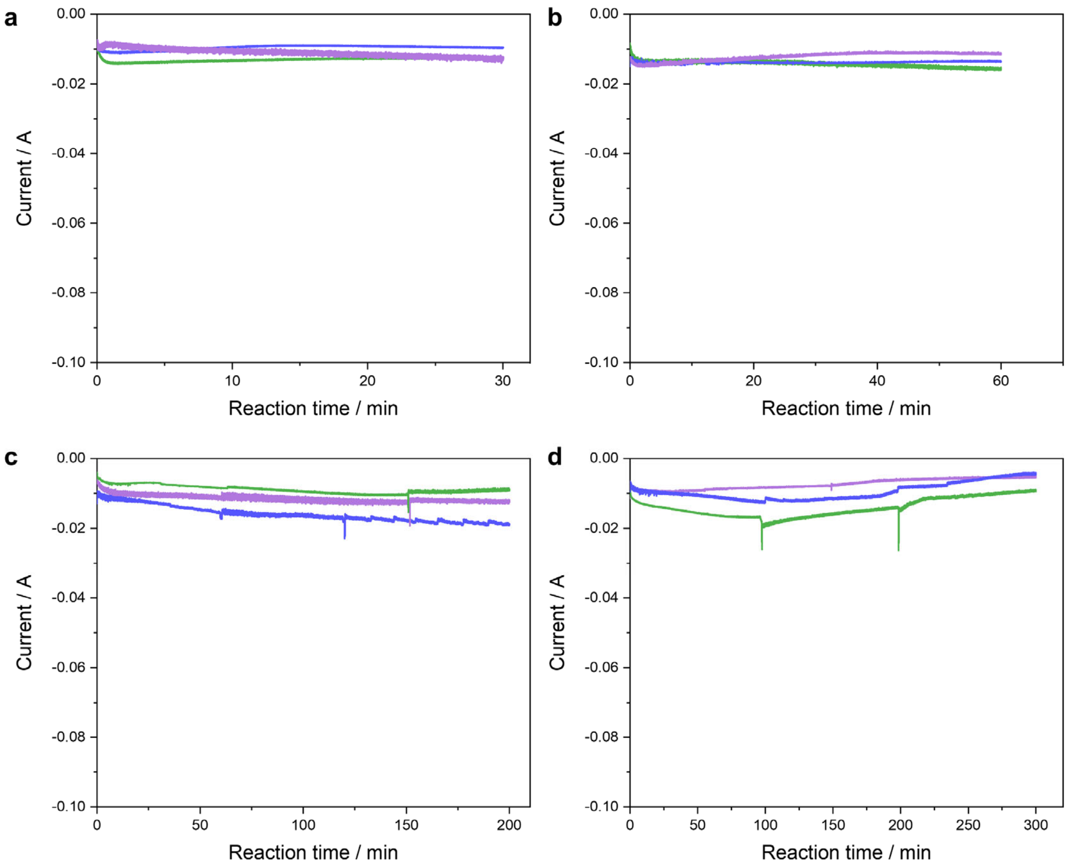

Supplementary Fig. 47. The chronoamperometry profiles of different Cu SACs in 0.5 M $KHCO_3$ electrolyte at an applied potential of −1.2 V vs RHE. (**a**) Cu/C_3N_4 for 30 min. (**b**) CuPc for 60 min. (**c**) Cu-NC for 200 min. (**d**) Cu-SNC for 300 min. The green, blue, and purple curves show the chronoamperometry profiles in three parallel experiments.

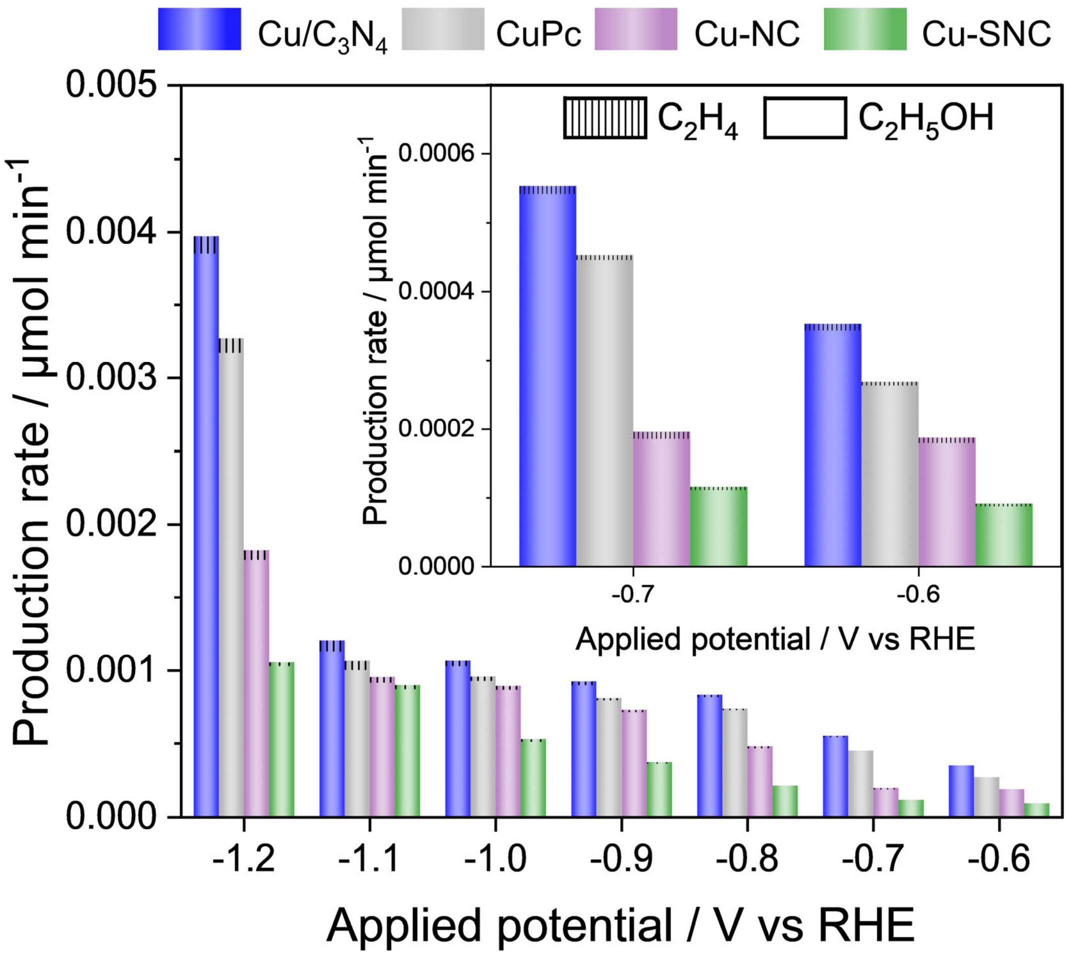

Supplementary Fig. 48. The evolution rates of Cu/C_3N_4, CuPc, Cu-NC, and Cu-SNC SACs from *operando* SEIRAS measurements. The inset shows the production rates of C_2H_4 and C_2H_5OH on Cu SACs at −0.7 V and −0.6 V, respectively.

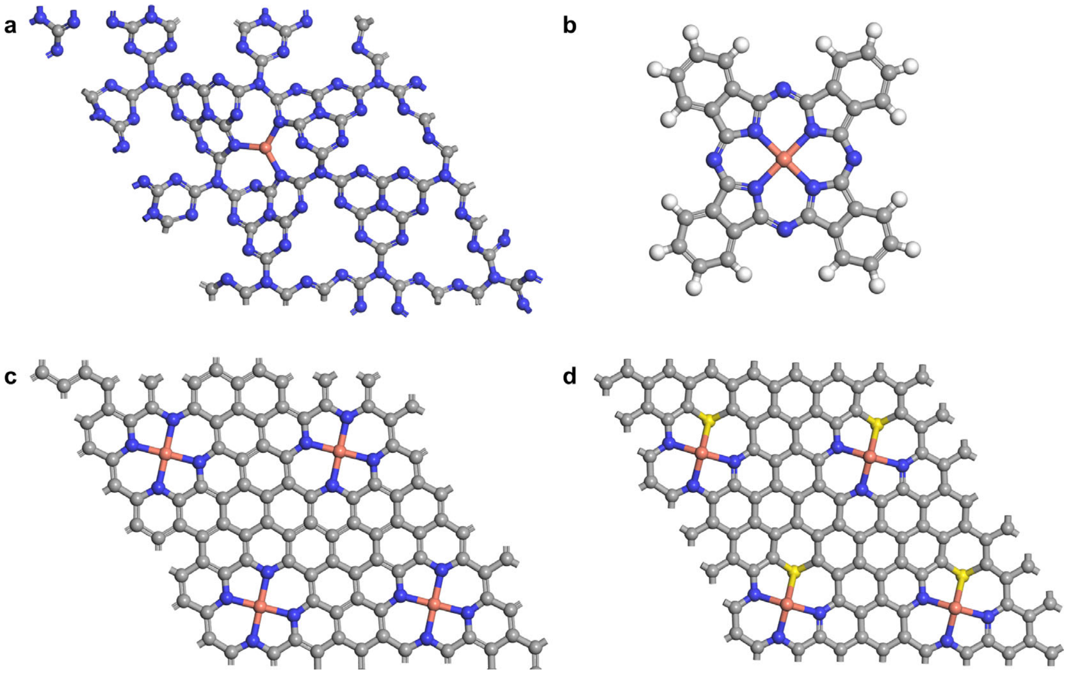

Supplementary Fig. 49. The optimized structures of the Cu SACs. (**a**) Cu/C_3N_4, (**b**) CuPc, (**c**) Cu-NC, and (**d**) Cu-SNC.

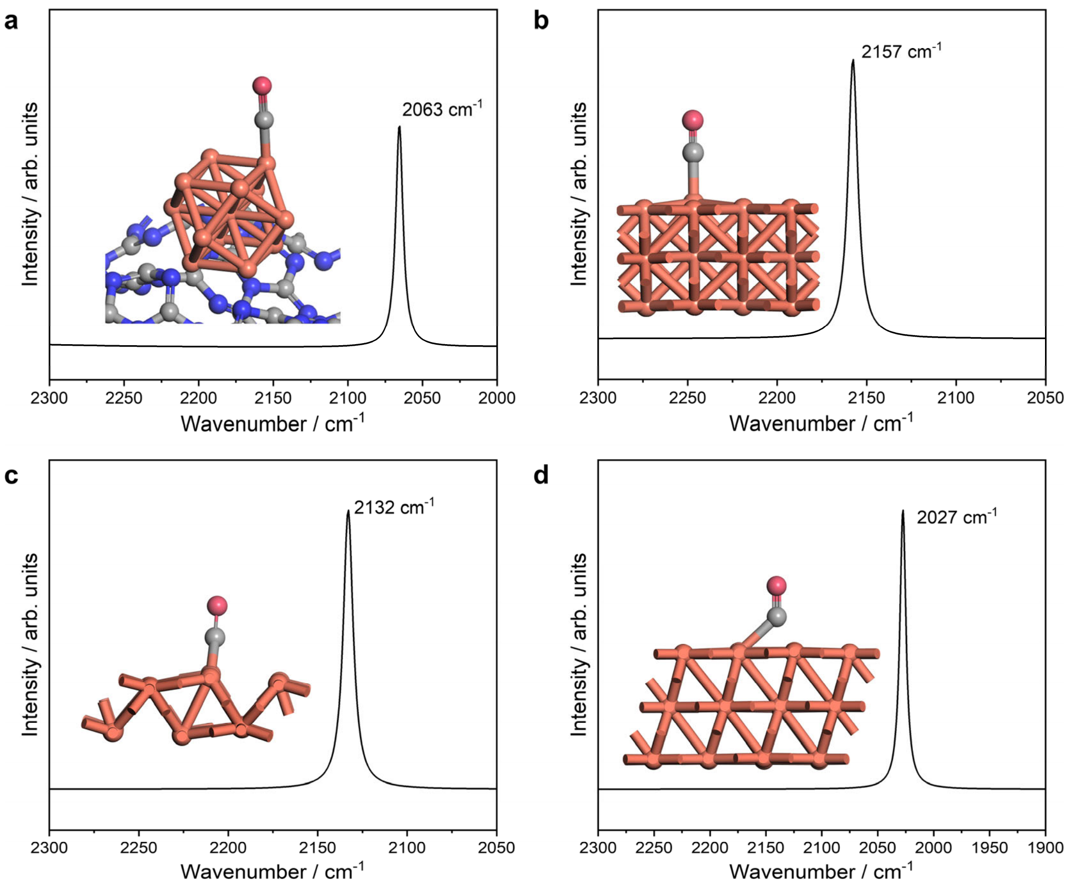

Supplementary Fig. 50. Simulated infrared spectra of CO adsorption on different metallic Cu surfaces. (**a**) C_3N_4 supported Cu nanoparticles. (**b**) Cu(100) facet. (**c**) Cu(110) facet. (**d**) Cu(111) facet. The inset shows the corresponding geometric configuration of CO adsorption.

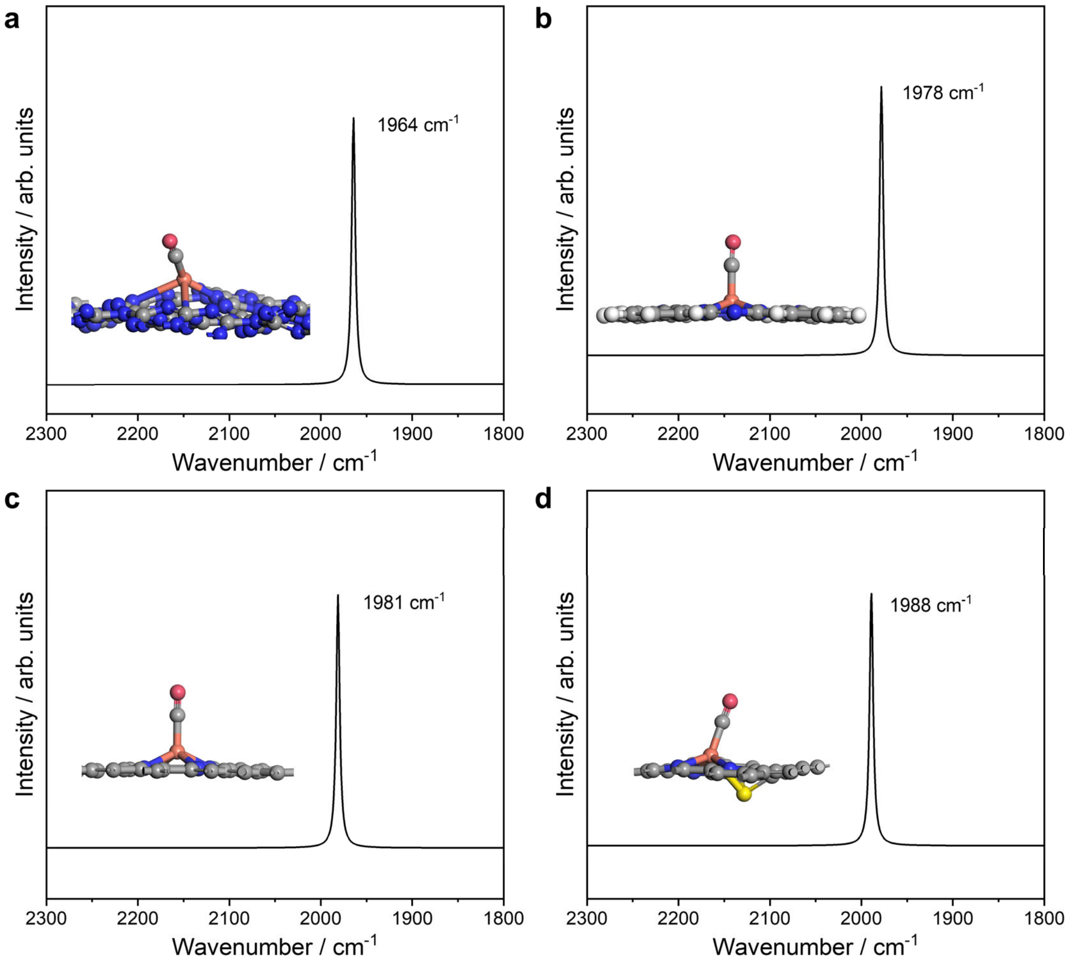

Supplementary Fig. 51. Simulated infrared spectra of CO adsorption on different Cu single sites. (**a**) Cu/C_3N_4 SACs. (**b**) CuPc SACs. (**c**) Cu-NC SACs. (**d**) Cu-SNC SACs. The inset shows the corresponding geometric configuration of CO adsorption.

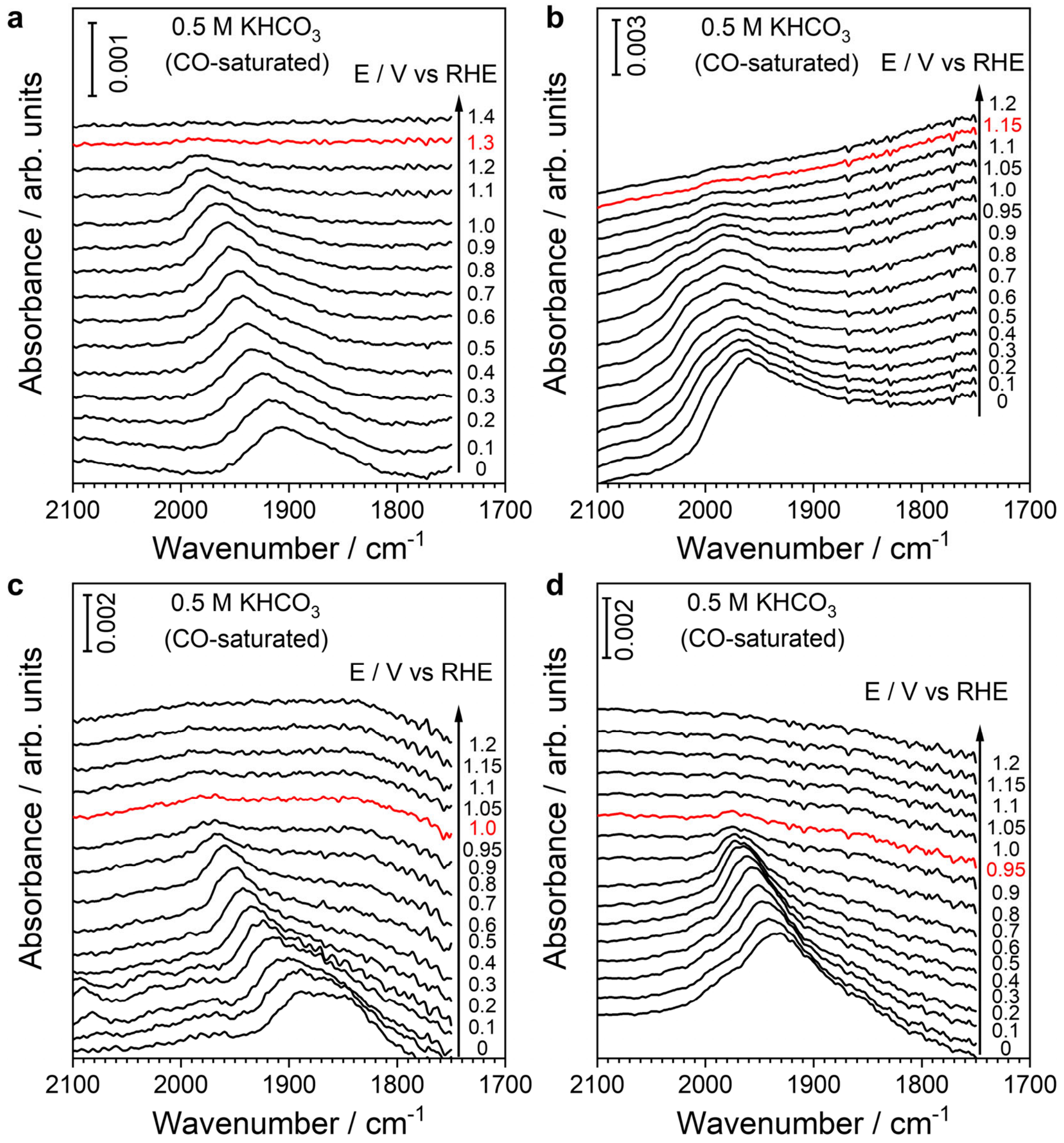

Supplementary Fig. 52. Potential-dependent SEIRA spectra of (**a**) Cu/C_3N_4, (**b**) CuPc, (**c**) Cu-NC, and (**d**) Cu-SNC SACs in CO-saturated 0.5 M $KHCO_3$ solution. The red curves show that CO adsorption disappears at a potential higher than 1.3, 1.15, 1.0, and 0.95 V for Cu/C_3N_4, CuPc, Cu-NC, and Cu-SNC SACs, respectively.

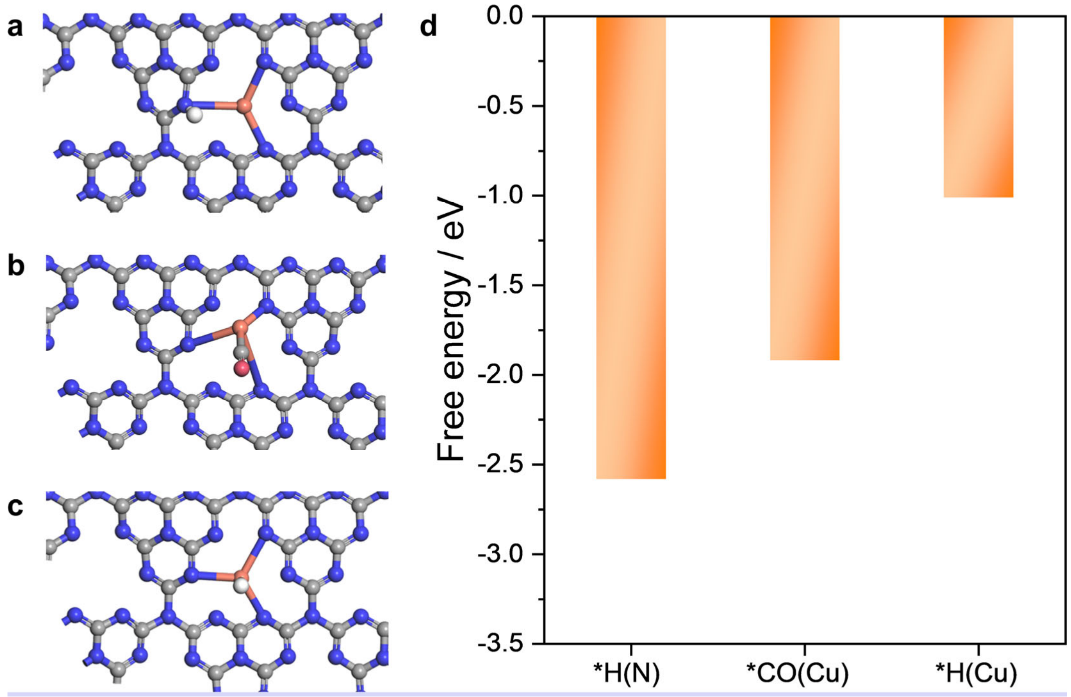

Supplementary Fig. 53. The configuration of (**a**) *H on N sites, (**b**) *CO on Cu single site, and (**c**) *H on Cu single site. (**d**) The corresponding free energy for the three adsorptions. The adsorption energy of *H on N site is the lowest, indicating that the *H on the N site is most likely to promote the breaking of Cu-N bonds.

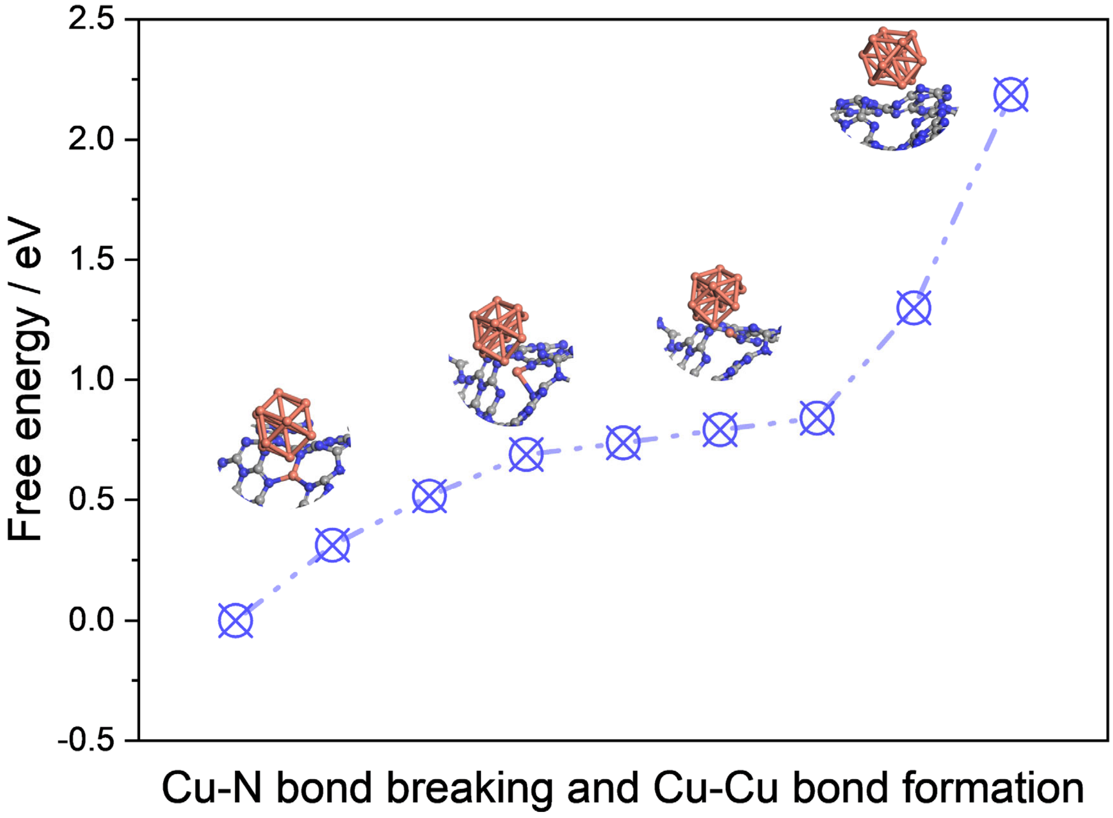

Supplementary Fig. 54. Free energy of leaching a Cu atom from Cu/C_3N_4 SACs. The transient Cu nanoparticle is instable which would further induce the breaking of Cu-N bonds. Due to the high computational cost, the applied potential is not considered during the calculations.

11. Cu Content and EXAFS Fitting Parameters for Cu SACs

Supplementary Table 1. ICP-OES results for the determination of Cu contents in Cu SACs.

Cu SACs	Cu wt%
Cu/C_3N_4	0.47
Cu-NC	0.76
Cu-SNC	0.30

Supplementary Table 2. Structural parameters extracted from the Cu K-edge EXAFS fitting.

Sample	Scattering pair	CN	R (Å)	σ^2 (Å^2)	ΔE_0 (eV)	R factor
Cu/C_3N_4	Cu-N	2.8	1.93	0.009	2.0	0.005
CuPc	Cu-N	4.0	1.97	0.003	6.2	0.007
Cu-NC	Cu-N	4.1	1.98	0.009	7.6	0.003
Cu-SNC	Cu-N	3.1	1.96	0.008	7.4	0.007
	Cu-S	0.8	2.45	0.046		

CN represents the coordination number. R is interatomic distance. σ^2 is Debye-Waller factor. ΔE_0 is edge-energy shift. R factor indicates the goodness of fit. The accuracies of the structural parameters are estimated as N ± 20%; R ± 1%, σ^2 ± 20%, and ΔE_0 ± 20%. The fitting ranges of k and R are 2.0−10.1 Å^{-1} and 1.0−1.9 Å for Cu/C_3N_4 SACs. The fitting ranges of k and R are 3.3−12.5 Å^{-1} and 1.0−1.9 Å for CuPc SACs. The fitting ranges of k and R are 2.0−11.1 Å^{-1} and 1.0−1.9 Å for Cu-NC SACs. The fitting ranges of k and R are 3.0−11.7 Å^{-1} and 1.0−2.6 Å for Cu-SNC SACs.

Supplementary Reference

1. Yang, X., Nash, J., Oliveira, N., Yan, Y. S. & Xu, B. Understanding the pH dependence of underpotential deposited hydrogen on platinum. *Angew. Chem. Int. Ed.* **58**, 17718−17723 (2019).
2. Jiang, T. *et al*. Trends in CO oxidation rates for metal nanoparticles and close-packed, stepped, and kinked surfaces. *J. Phys. Chem. C* **113**, 10548−10553 (2009).
3. Nørskov, J. K., Hammer, B. & Morikawa, Y. CO Chemisorption at Metal Surfaces and Overlayers. *Phys. Rev. Lett.* **76**, 2141 (1996).
